# Piloting cannabis for sleep disturbance in palliative care over 5 weeks: The pillow study protocol

**DOI:** 10.1371/journal.pone.0361190

**Published:** 2026-11-11

**Authors:** Alison M. Newton, Jessica L. Krok-Schoen, Erin E. Stevens, Shanna L. Babalonis, Theodore L. Wagener, Alice Hinton, Roberto Benzo, Theodore M. Brasky

**Affiliations:** 1 Division of Medical Oncology, The Ohio State University College of Medicine, Columbus, Ohio, United States of America; 2 Division of Health Sciences, School of Health and Rehabilitation Sciences, The Ohio State University College of Medicine, Columbus, Ohio, United States of America; 3 Division of Palliative Medicine, The Ohio State University College of Medicine, Columbus, Ohio, United States of America; 4 Department of Behavioral Science, University of Kentucky College of Medicine, Lexington, Kentucky, United States of America; 5 Center for Tobacco Research, The Ohio State University Comprehensive Cancer Center, Columbus, Ohio, United States of America; 6 Division of Cancer Prevention and Control, The Ohio State University College of Medicine, Columbus, Ohio, United States of America; University of Texas at Austin, UNITED STATES OF AMERICA

## Abstract

**Background:**

Sleep disturbance affects 60% of cancer patients, 3-fold that of the general population, and is frequently driven by clinical characteristics, physical symptoms, and psychological distress. Sleep disturbance and other symptoms often exacerbate one another, leading to reduced health-related quality of life (HRQOL). Sleep is the most commonly reported reason for cannabis use among adults with cancer, yet clinical evidence supporting its use for symptom management remains limited.

**Objectives:**

This report describes a protocol for a pilot randomized trial designed to evaluate the safety and feasibility of a cannabis-based intervention and to explore its preliminary efficacy for sleep disturbance and HRQOL.

**Methods:**

This pilot parallel-arm randomized clinical trial will enroll 50 patients from the Ohio State University Comprehensive Cancer Center (OSUCCC; anticipated Fall 2026). Eligible participants will be new to the OSUCCC’s Palliative Care clinic and report at least moderate sleep disturbance. Participants will be randomized by sex and race strata to receive either usual care or a nightly oral cannabis gummy containing 5 mg Δ-9-tetrahydrocannabinol (THC) and 5 mg cannabidiol (CBD), taken 1 hour before bedtime for 30 days. Adverse events will be monitored throughout the study. Sleep, HRQOL, and related symptoms will be assessed using self-reported questionnaires, ecological momentary assessments, and actigraphy.

**Conclusions:**

Findings will provide critical safety, feasibility, and preliminary efficacy data to support a future large-scale, multisite trial targeting sleep disturbance and symptom palliation in cancer care.

## Introduction

Sleep disturbance occurs in 60% of cancer patients [1], three times higher than in the general population [2]. In adults with cancer, sleep disturbance may be caused by multiple—often co-occurring—factors including clinical characteristics (e.g., cancer site and stage), physical complaints (e.g., pain), and psychological distress [3,4]. Sleep disturbance is associated with numerous negative outcomes in cancer patients, especially reduced health-related quality of life (HRQOL) and emotional distress [2,5–7]. Disruptions in sleep manifest as a vicious cycle with other symptoms such that significant symptom burden can contribute to sleep disturbance, and sleep disturbance may further compound symptom burden.

In the past two decades, use of cannabis products among adults has increased nearly 200% [8–11], particularly as an adjunct to treat pervasive symptoms amid changing legal frameworks [12,13]. While the acceptability of cannabis use has grown [14,15], and medical and non-medical cannabis programs have expanded at the state level [16,17], the specific effects of cannabis on sleep disturbance and other cancer-related symptoms has not been comprehensively studied in cancer populations. The primary cannabinoids found in cannabis products, including Δ-9-tetrahydrocannibinol (THC) and cannabidiol (CBD), may promote sleep by increasing sedation, decreasing sleep latency, and inhibiting cortisol release [18,19]. There is limited experimental evidence supporting cannabis for sleep outcomes in cancer patients and existing data are restricted to secondary outcomes in patients without an initial sleep complaint [20–26]. Further, most studies have utilized pharmaceutical preparations of cannabis which do not reflect how cannabis is used in the real world [20–26]. It is therefore critical to better understand the potential benefits and risks of cannabis for alleviating sleep disturbance and other symptoms in cancer patients.

To address this gap, a five-week randomized trial will be conducted to assess the effects of a nightly, low dose cannabis gummy (containing 5 mg THC and 5 mg CBD) versus usual care, among 50 adults with cancer at the Ohio State University Comprehensive Cancer Center (OSUCCC). The overarching objective is to determine the safety, feasibility, and compliance with this nightly cannabis product, and to provide preliminary efficacy data on symptom burden to inform future work.

## Materials and methods

### Design overview

This pilot randomized, parallel-arm trial will examine patient safety, feasibility, compliance, and preliminary efficacy of a cannabis gummy versus usual care in 50 new cancer patients to the OSUCCC’s Palliative Care clinics who report sleep disturbance and have not used cannabis in the past 6 months (Fig 1). As this is a pilot trial, its design is unblinded and unbalanced. Detailed eligibility screening procedures, including biometric screening, will be conducted. Participants will be randomized to either a nightly dose of a cannabis gummy for 30 days or usual care; participants subsequently answer a baseline questionnaire and will be provided with an ActiGraph device to be worn every day for the duration of the study. Weblinks to brief ecological momentary assessments (EMAs) will be sent via text message or email three days/week to record adverse events (AEs), blood pressure (via take-home monitor), crossover events (i.e., use of sleep aids or cannabis, as applicable), sleep disturbance, and HRQOL. On Day 31, patients will complete a comprehensive follow-up questionnaire to assess AEs, symptomology, and medication use as well as biometrics. One week following the end of the intervention period, patients will be sent a brief closeout questionnaire (Fig 2). Each participant is enrolled for approximately 38 days.

**Fig 1:**
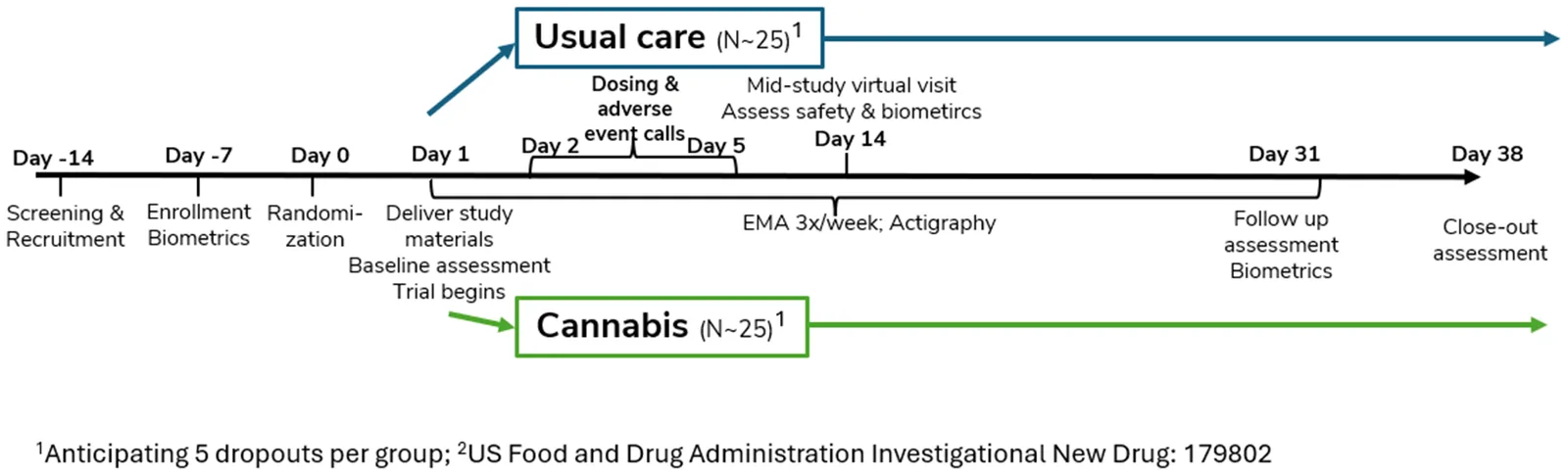
Study design. Fifty participants will be recruited into a randomized clinical trial (Days −14 to −7) and, upon confirmation of eligibility, randomized to the cannabis intervention or usual care (Day 0). Participants will wear an ActiGraph device for the duration of the trial and answer ecological momentary assessment questionnaires three days/week. Follow-up assessments and biometrics will be completed on Day 31 and a close-out assessment on Day 38.

**Fig 2:**
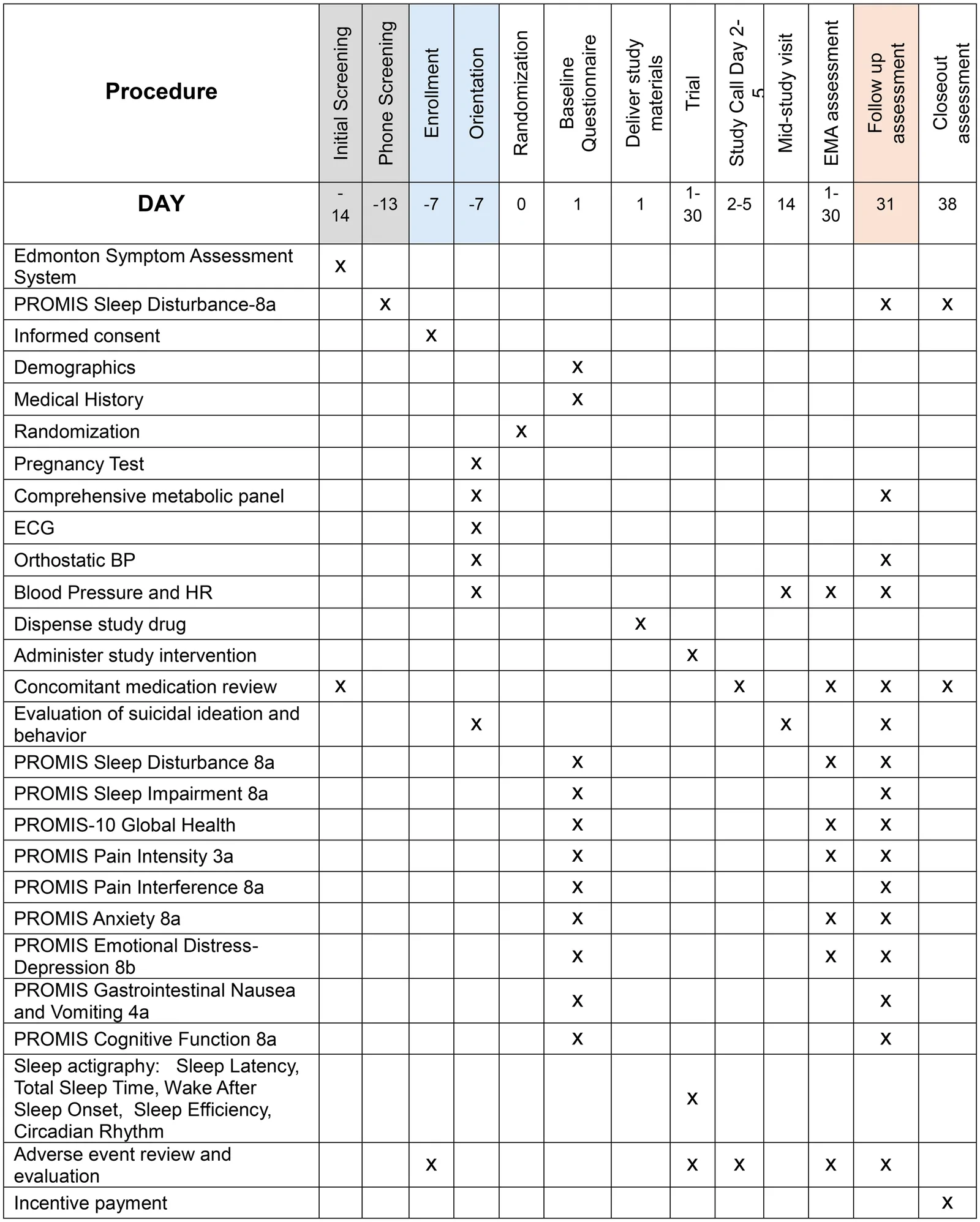
Study schedule of events.

### Objectives and hypotheses

The trial will generate safety, feasibility, and preliminary efficacy data to support a large-scale clinical trial of cannabis for sleep disturbance and symptom palliation. The aims of the trial are to 1) determine the safety and feasibility of a nightly cannabis gummy versus usual care, and 2) examine the preliminary efficacy of the intervention on sleep disturbance and HRQOL, among palliative care cancer patients.

In those randomized to the cannabis arm will, it is hypothesized that they will: 1) safely tolerate the cannabis (defined as no treatment-related serious adverse events, similar rates of Grade I-III adverse events compared with usual care); 2) demonstrate high rates of engagement, defined as ≥80% adherence to the study protocol and ≥80% retention over the trial duration; and 3) experience improved sleep and HRQOL at follow-up compared to usual care.

### Planned recruitment

This trial will enroll 50 cancer patients new to the OSUCCC’s Palliative Care clinics who report sleep disturbance between October 1, 2026, and September 30, 2027. Palliative Care providers will identify patients with sleep disturbances from an in-clinic Edmonton Symptom Assessment System (ESAS) screener. Patients who report moderate to severe drowsiness (ESAS ≥4) will be referred to the study team for preliminary eligibility review based on criteria found in their medical record (Table 1). Upon passing the preliminary review, research staff will contact patients via phone to complete an eligibility screener. Patients endorsing “poor” or “very poor” sleep in the past week on the first item of the Patient-Reported Outcomes Measurement Information System (PROMIS) Sleep Disturbance-Short Form 8a, self-reported no cannabis use in the past 6 months, no medications contraindicated with medical cannabis, no sleep aid usage, and pass all other health history questions will be deemed preliminarily eligible (Table 1). Patients who express interest in the study will be invited to an in-person screening visit, consisting of written informed consent and biometric screening procedures.

**Table 1:** Inclusion and exclusion criteria.

Inclusion Criteria	
1.	New patients in the OSUCCC’s Palliative Care clinics who have histologically or cytologically confirmed malignancy of any anatomic site. This applies to either newly diagnosed cancer or those with preexisting malignancy receiving treatment.
2.	Reported sleep disturbance (≥4) in the Edmonton Symptom Assessment System
3.	Patients with active cancers and are under any line of treatment or have received cancer treatment in the past 6 months
4.	If patients are currently receiving cancer therapy, they must have been taking their current anti-cancer intervention/ therapy regimen for at least one month prior to enrollment (to ensure no safety or toxicity issues with study drug initiation).
5.	Age ≥ 18 years.
6.	English speaking with the ability to understand and the willingness to sign a written informed consent document.
7.	Eastern Cooperative Oncology Group performance status ≤3
8.	Adequate organ and marrow function within the past 3 months or predating their current cancer regimen defined: • total bilirubin: < 3X institutional upper limit of normal (1.5 mg/dl) • AST(SGOT)/ALT(SGPT): < 3X institutional upper limit of normal • AST: < 3X institutional upper limit of normal (10–39 U/L) • ALT: < 3X institutional upper limit of normal (10–52 U/L) • glomerular filtration rate (GFR): ≥ 30 mL/min/1.73 m2 using Cockcroft-Gault • normal EKG; or non-normal EKG with no significant arrhythmias or severe CHF. A normal EKG defined as sinus rhythm with no ST or T wave changes any clinically concerning EKGs will be reviewed by the study physician to determine participant eligibility into the study
9.	Patients must be on a stable dose of pain medication for two weeks and not requiring rapid dose escalation.
10.	Individuals able to become pregnant will agree to practice a highly effective form of birth control.
11.	Agree to study requirements, as described in the consent form: a. no driving, use of heavy machinery, or caring for children for 12 hrs after dose b. wearing fitness tracker 24/7 for the duration of the study c. no cannabis (i.e., outside of provided study drug), or illegal drug use d. calls so that study staff can monitor dosing e. completing weekly EMA questionnaires
Exclusion Criteria	
1.	History of hypersensitivity to cannabis, THC, or CBD
2.	Current, regular use of cannabis/marijuana, THC-containing prescription medications, prescription CBD, or over-the-counter CBD oil within the past 6 months
3.	Concurrent use of medications that are contraindicated with medical cannabis: valproate, clobazam, warfarin, fluoxetine, disulfiram, tamoxifen, Ibrance, Gleevec, amphetamines, buprenorphine, methadone, alprazolam, amiodarone, fluconazole, ketoconazole, itraconazole, clarithromycin, ritonavir, erythromycin. The study physician will review eligibility for participants taking medications that are moderate to strong inhibitors and inducers of enzymes CYP3A4, CYP2C19, and CYP2C9.
4.	The following medications if being used to treat sleep: benzodiazepines or nonbenzodiazepine medications, antidepressants, antihistamines, supplements (e.g., melatonin)
5.	Cardiac conditions contraindicated for cannabis use, moderate/severe or unstable CHF, symptomatic valvular heart disease, and uncontrolled arrhythmias.
6.	Abnormal screening ECG with corrected QT (QTc) intervals of ≥450 (males) and ≥460 (females)
7.	Baseline orthostatic hypotension drop of BP ≥ 20 mmHg, or in diastolic BP of ≥10 mmHg or experience of lightheadedness or dizziness during the test
8.	Based on a measurement at baseline, participants with current hypertension defined as BP > 165/100, abnormal ECG results or a resting heart rate defined as >110bpm.
9.	Concomitant use of checkpoint inhibitors
10.	Current use of investigational agents or <3 months after the use of investigational agents
11.	Diagnosis of sleep apnea
12.	Allergy to any constituent/ingredient contained in the edible doses
13.	Psychiatric illness/social situations that would limit adherence with study requirements (e.g., bipolar disorder, psychosis). Mild/moderate mood disorders treated by medications that are not expected to increase cannabis-induced sedation/impairment are acceptable with physician approval.
14.	Any known or suspected history or family history of schizophrenia, bipolar disorder, or other psychotic illness
15.	Pregnant or breastfeeding
16.	Sole caretaker of minor children living in the household
17.	History of seizure disorder, epilepsy (controlled or uncontrolled)
18.	Current legal obligations (parole, probation, incarceration)
19.	Current substance use disorder (including cannabis use disorder), or currently enrolled in substance use treatment
20.	Self-reported cannabis and synthetic cannabinoid use in the past 6 months
21.	Self-reported illicit drug use in past 60 days or self-reported daily alcohol use
22.	No access to internet/data or devices needed to participate in video calls (day 14) and EMA assessments

### Procedures

#### Orientation and randomization.

Individuals who pass the initial eligibility phone screeners will be invited to the OSU Center for Tobacco Research to complete an in-person screening visit. Participants will have time to discuss the study, ask questions, and if still interested, provide written informed consent, obtained by trained study staff. Once consented, participants will complete biometric screening procedures to finalize eligibility into the study. Trained and certified research staff will collect vitals (blood pressure, heart rate, orthostatic blood pressure), an electrocardiogram (ECG), and a blood draw for a comprehensive metabolic panel. Research staff will additionally explain the actigraphy device and consumer-grade wrist blood pressure monitor to participants and give a brief tutorial on their use. Detailed dosing, storage, security, and administration instructions for the cannabis gummy will be provided as well. The research team will describe in detail the proper dosing escalation procedure for the cannabis gummy and describe the phone assessments with research staff. Participants will be given phone numbers and instructions on how to contact a medical provider should an urgent adverse event occur.

A participant with adequate comprehensive metabolic panel numbers, ECG, baseline orthostatic hypotension, and baseline vitals (detailed in Table 1) on the biometric screening will be considered eligible for the trial, upon which they will be randomized within small randomly-sized blocks stratified by sex and race (white/non-white) to either a nightly dose of a cannabis-based gummy for 30 days or usual care. While we are aware of data suggesting sex differences in AE reporting in a recent trial of higher doses (7.5 mg-10 mg *bis in die*) of oral cannabis [27], we know of no data to indicate that sex or race might affect the experience of AEs or their reporting, or otherwise affect study feasibility, at the doses we are studying. We chose to stratify randomization procedures for two reasons: 1) we wanted to ensure balance across the groups based upon major sociodemographic features given the study’s meager sample size, and 2) prior data including data from the OSUCCC (unpublished) suggesting sex differences in associations of cannabis use with patient-reported symptoms including sleep, our preliminary outcome [28]. Unblinded stratified, blocked randomization will be conducted using the Research Electronic Data Capture (REDCap) platform by the study biostatistician who will have access to the allocation sequence. Usual care: Participants randomized to the usual care arm will continue to receive routine care at the OSUCCC Palliative Care clinics. Routine care may include prescription medications to alleviate pain or emotional distress, which may also affect sleep. Usual care may include, but not will not be limited to, prescriptions of non-habit-forming sleep aids (including melatonin, anxiolytics, and antidepressants) at the discretion of their provider. Habit-forming prescription hypnotics are rarely prescribed. The choice of a usual care comparator was selected due to the size and scope of the pilot trial including practical, financial, and regulatory constraints. Intervention: Participants randomized to the intervention arm will continue to receive routine care. Palliative care providers will be permitted to prescribe medications for pain or emotional distress as needed; however, they will be asked to avoid treating sleep directly. Instead, all patients assigned the intervention will be asked to self-administer a nightly dose of a cannabis gummy one hour prior to bedtime.

#### Study drug and dose escalation procedures.

Two doses of cannabis gummies (2.5 mg THC + 2.5 mg CBD; 5 mg THC + 5 mg CBD) will be obtained (ElSohly Laboratories, Inc). The OSU Investigational Drug Service (IDS) will store and prepare doses for each participant randomized to the intervention. On Days 1–4, participants will consume the lower cannabis dose (2.5 mg THC + 2.5 mg CBD). Study staff will contact patients on the mornings of Days 2–5 to assess the presence and severity of AEs. If any of the initial escalation doses (Days 1–4) result in treatment-related AEs, research staff will contact the study medical advisor who will determine whether the participant should withdraw from the trial. If the escalation doses were safely tolerated, participants will take the full dose (5 mg THC + 5 mg CBD) on the evening of Day 5. Participants unable to tolerate the full dose gummy will be withdrawn from the study and will be considered to have experienced an AE. Due to this small pilot trial’s size, scope, and limited resources, participants are unable to stay on trial with a dose reduction. Participants will be asked to keep the study medication refrigerated for the study duration and report any deviations from the storage, dosing, or administration to research staff.

#### Baseline.

On Day 1, study participants will receive their study materials including the activity monitor, the blood pressure monitor (intervention arm) and the 30-day supply of study drug (intervention arm). All participants will complete an electronic baseline questionnaire focused on their cancer symptoms, demographics, and ideation of self-harm. Participants randomized to the intervention will begin their cannabis dosing one hour before bed in the evening.

#### Days 2–5, dose escalation (intervention group only).

On Days 2–5, research staff will call participants on the cannabis intervention arm to assess AEs and determine the tolerability of the initial doses. On Day 5, the medical/research team will determine if the participant should remain at 100% of the target dose or be withdrawn from the study. Research staff will schedule Day 14 visit with participants at this time.

#### Days 1–30.

Participants will receive weblinks to brief EMAs via email or text message three days/week to record AEs, cannabis dose taken, sleep quality, blood pressure, heart rate, and symptoms.

#### Day 14 (intervention group only).

Research staff will conduct a video call with participants on the cannabis intervention to assess safety/AEs. Participants will be asked about any new or worsening side effects and assess ideation of self-harm. Participants will check their vitals while on this call and report the values to the study staff. Research staff will also assess adherence to study procedures.

#### Day 31.

Participants will complete a follow-up electronic questionnaire to report symptoms, ideation of self-harm, and medication use. Participants will also return the ActiGraph device, blood pressure monitor, and remaining cannabis product (if any remaining). Remaining product will be collected and destroyed by the research pharmacy. Intervention group participants will complete a follow-up biometric assessment that includes a comprehensive metabolic panel via blood draw, orthostatic hypotension, and vitals. If orthostatic hypotension values are flagged or the patient is symptomatic, we will contact the study’s medical advisor for guidance.

#### Day 38.

One week following the end of the intervention period, patients will complete a brief electronic closeout questionnaire. The final questionnaire will assess their overall impressions of cannabis use, sleep disturbance (PROMIS Sleep Disturbance Short-Form 8a), presence of rebounding or cannabis withdrawal symptoms, and post-trial use of cannabis or sleep aids.

### Participant incentives

On Day 38, participants will receive monetary compensation via ClinCard for their participation in the baseline study visit, the completion of study assessments, and regular use of the ActiGraph device. Participants will be reimbursed for their travel for baseline and follow-up visits as well. Total compensation will be up to $200 per patient.

### Ethical declarations

We have obtained all necessary approvals including United States Food and Drug Administration Investigational New Drug approval (Number: 179802), Institutional Review Board approval (Study Number 20252840, without modifications approved on 5/22/2026), United States Drug Enforcement Agency Schedule I licensure (granted 7/31/2026). This study is registered with Clinicaltrials.gov under the identifier NCT07658677, submitted 6/22/26. This study has received funding through an internal grant from OSUCCC’s Pelotonia IDEA Award. All participants recruited for this trial will be informed of their rights as research participants and will be provided with as much time as necessary to provide informed consent. Study staff will review the consent form with the participant and assess the individual’s understanding of the consent form and required study procedures. To protect the privacy of participants, study information will be collected in a private setting by phone to reduce likelihood of personal, medical, or sensitive information being overheard. Also, data will be de-identified to increase privacy and decrease the risk of any breach of confidentiality. All study modifications to the protocol will be submitted to institutional ethics panels and ClinicalTrials.gov.

### Primary outcomes

Endpoints will be evaluated based on safety and feasibility (i.e., adherence and retention): Safety will be evaluated by assessing the frequency, grade, and type of AE reported by participants. The frequency and percentage of participants experiencing each grade of AE on Day 30 will be reported for both groups. Adherence will be measured as the proportion of participants who adhere to the study protocol, which includes consistent use of actigraphy, completion of self-reported measures, and measurement of self-reported cross-over events. Retention will be the proportion of participants who remain in the study after consent and randomization and complete all study assessments by Day 38. Adherence (including <20% crossovers) and retention will each be considered successful at ≥80%.

### Secondary outcomes

The study’s secondary outcomes are exploratory and will provide initial insights into the intervention’s potential efficacy. Preliminary analyses will examine the effect of cannabis on sleep disturbance and HRQOL between follow-up measures. Secondary endpoints (e.g., actigraphy, EMA) will be assessed to explore cannabis’ broader effects.

### Analysis

We anticipate data collection completion after one year of recruitment and data analysis will begin shortly after. All analyses will be completed under an intention-to-treat framework. Safety will be evaluated by assessing the frequency, grade, and type of AE reported by participants. The frequency and percentage of participants experiencing each grade of AE will be reported for both groups. Comparisons of AE incidence between the intervention and control groups will be conducted using Fisher’s exact test. Feasibility will be evaluated based on adherence, crossover, and retention. Adherence rates will be analyzed using descriptive statistics. Logistic regression will be used to identify demographic and clinical factors associated with adherence and retention. While statistical tests will be conducted, the focus will be on the magnitude, direction, and precision of observed differences.

Given the exploratory nature of the second aim, formal hypothesis testing will not be conducted. Instead, the objective is to present changes in these outcomes from baseline to Day 31 to provide initial insights into the intervention’s potential efficacy. Preliminary analyses will examine the effect of cannabis on sleep disturbance and HRQOL using means, standard deviations, and confidence intervals between follow-up measures. Secondary endpoints (e.g., actigraphy, EMA) will be assessed to examine cannabis’ effects. We will compare patient demographics and intervention status between those who completed the study and those lost to follow-up to check for missing data patterns. Primary analyses will be performed using all available outcome data; no imputation or other procedures to account for missing data will be employed. As we expect end of study PROMIS measures to be missing at random, the estimates generated from the proposed likelihood based procedures will be unbiased with this approach. We anticipate study manuscripts to be submitted for peer review by October 2028.

### Sample size and power

For this pilot trial, we will recruit 50 patients (25 per group), with an anticipated attrition rate of 20%. This sample size was selected to provide useful estimates of safety, tolerability, and feasibility parameters and their variability to inform the design of a subsequent trial. We anticipate that 50 patients will allow estimation of feasibility and adherence (set at ≥80%, as described above) and to characterize their uncertainty. The sample size also represents a practical sample to characterize the frequency, severity, and type of adverse events, given the planned recruitment period and resources required to provide and monitor the study intervention. As our efficacy aims are preliminary, no formal power calculations were used. The data collected will be used to refine the intervention and inform the design and power calculations for future trials.

### Data management

All study materials, survey data, clinical data (including AEs, concomitant medications, and expected adverse reactions data) and clinical laboratory data will be entered into a 21 CFR Part 11-compliant data capture system. The data system includes password protection and internal quality checks, such as automatic range checks, to identify data that appears inconsistent, incomplete, or inaccurate. Participants will be assigned a unique study identification number (study ID). All study data will be identified by study ID only. Any electronic information will only be accessible to authorized study personnel. Given the size of this pilot trial, a data monitoring committee will not be established.

### Safety considerations: Potential risks and risk mitigation

#### Cannabis.

Potential side effects from the use of cannabis include, but are not limited to, the following: dizziness, anxiety, confusion, sedation, changes in blood pressure, increases in heart rate, red eyes/conjunctiva, impairment of short-term memory, euphoria, difficulty in completing complex tasks, suppression of the body’s immune system, inability to concentrate, impaired motor skills, paranoia, psychotic symptoms, general apathy, depression, and/or restlessness. Although not expected, additional symptoms may include neurologic disorders, behavioral problems, neuropsychiatric effects, cardiovascular disorders, respiratory disorders and gastrointestinal disorders. Adverse events (AEs or serious adverse events) will be queried during study phone calls, EMAs, a study participant presenting for medical care, or upon review by a study monitor. The primary clinician will identify and record AEs and safety concerns throughout the course of the study and make decisions on participant continuation or withdrawal from the study. The type, severity, and frequency of AEs will be collected to identify and characterize any safety concerns that may arise. Participants will be discharged from the study if they experience a SAE that is possibly, probably or definitely related to the study medication. The study will be stopped, and a safety evaluation will be conducted if two participants experience SAEs that are possibly, probably or definitely related to the study medication.

#### Confidentiality and privacy.

There is a minimal risk of loss of confidentiality in this study. Precautions will be taken to protect electronic data from unauthorized disclosure, tampering, or damage by controlling access to the computers and files that hold this information. Access to computer files will only be obtained through passwords and will only be accessed by Institutional Review Board-authorized research study personnel. Only authorized research study personnel will have permission to examine electronic records to identify which patients are eligible to approach.

#### Other risks.

There are risks of drug diversion and accidental ingestion. To mitigate these risks, our research team will use two different types of medication locking procedures. First, the interventional drug will be placed in child-proof and tamper-proof locking pill vials (SafeRx Pharmaceuticals^®^, Lenox, MA). Access codes will be provided to participants at the start of each week. Second, weekly pill vials will be placed in a waterproof bag with a combination lock on the zipper. The access code will be provided to the participants at the start of each week.

### Data sharing and dissemination

De-identified individual-participant level data will be made available upon reasonable request for purposes of replication. All shared data will be appropriately de-identified through the removal of personally identifiable information prior to dissemination, and these data sharing procedures will be clearly described in the informed consent documents. To support transparency, reproducibility, and secondary analyses, comprehensive data dictionaries and codebooks will be developed. The study team’s dissemination strategy is designed to reach researchers, clinicians, study participants, and the broader public. Findings from this trial will be presented at national scientific meetings and published in peer-reviewed journals. Study results will also be reported in a timely manner on ClinicalTrials.gov in accordance with applicable reporting requirements.

## Discussion

The proposed PILLOW trial will be the first to examine the preliminary effects of cannabis on sleep disturbance and HRQOL among cancer patients with reported poor sleep quality. This trial has several strengths; 1) it will be the first specifically designed to examine the efficacy of cannabis for sleep disturbance in cancer patients; 2) the first to utilize actigraphy to objectively measure sleep outcomes in this population; and 3) the study will be the first to utilize EMA to complement traditional baseline and follow-up assessments for symptom assessment in this context. There are also limitations of note. Chief among them, is the trial’s relatively small sample size, which limits our ability to determine cannabis effects on patient-reported outcomes. This study’s primary objective is to inform safety and feasibility, and to aid in design and power considerations for a future trial. Further, the study’s generalizability is constrained by recruitment from individuals in a palliative care population at a single institution. However, OSUCCC is the third largest comprehensive cancer center in the country, and its catchment area includes the entire state of Ohio, thus providing a large potential participant population and ample resources. The trial is further limited by its unblinded design, which may increase the potential for expectation and reporting biases to influence study outcomes, including both AEs and patient-reported outcomes. Lastly, because of the limited resources, participants who cannot tolerate the intervention will be withdrawn rather than continued at a lower dose. This may limit our ability to characterize the feasibility and potential effects of lower-dose cannabis among participants who do not tolerate the target dose. This is the first study of its kind and will provide the foundation for a multi-site research program and critically needed pilot data on the safety and feasibility of a cannabis-based trial in cancer patients to inform future experimental work for symptom management in this population.

## Supporting information

S1 Checklist SPIRIT 2025 editable checklist. SPIRIT 2025 checklist of items to address in a randomized trial protocol.
(DOCX)

S1 File IRB protocol. (DOCX)

## Abbreviations

AE: Adverse Event
CBD: Cannabidiol
ECG: Electrocardiogram
EMA: Ecological Momentary Assessment
ESAS: Edmonton Symptom Assessment System
FDA: Food and Drug Administration
HRQOL: Health-Related Quality of Life
IDS: Investigational Drug Service
IND: Investigational New Drug
IPD: Individual Participant Data
OSU: Ohio State University
OSUCCC: Ohio State University Comprehensive Cancer Center
PROMIS: Patient-Reported Outcomes Measurement Information System
REDCap: Research Electronic Data Capture
SAE: Serious Adverse Event
THC: Δ-9-Tetrahydrocannabinol
