# Supplementary material for: Piloting cannabis for sleep disturbance in palliative care over 5 weeks: The pillow study protocol
Source: PLoS One. 2026 Nov 11;21(11):e0361190. doi: 10.1371/journal.pone.0361190 (PMC13754115; doi:10.1371/journal.pone.0361190)
Supplement: Supplementary file 2 [file pone.0361190.s002.docx]

**Piloting a novel therapeutic for sleep disturbance in palliative care over 5 weeks: The PILLOW Study**

**Principal Investigator:**	Theodore Brasky

College of Medicine, Medical Oncology

3650 Olentangy River Rd, Columbus OH 43214

614-293-3772

Theodore.Brasky@osumc.edu

**Drug/Device**

**Manufacturer**:	ElSohly Laboratories

5 Industrial Park

Oxford, Mississippi

**Initial version:**	V 1.0 [12/2/2025]

**Amended:**	V X [date]

**Table of Contents**

[**1. Abbreviations and Definitions**	3](#_Toc215649791)

[**2. Background and Rationale**	4](#_Toc215649792)

[**3. Objectives**	7](#_Toc215649793)

[**4. Study Design & Procedures**	8](#_Toc215649794)

[**5. Institutional Approvals**	13](#_Toc215649795)

[**6. Participant Population**	14](#_Toc215649796)

[**7. Cost to Participants and Incentives to Participate**	18](#_Toc215649797)

[**8. Compensation for Research-Related Injury**	18](#_Toc215649798)

[**9. Informed Consent Process**	19](#_Toc215649799)

[**10. Privacy of Participants**	20](#_Toc215649800)

[**11. Confidentiality and Management of Study Materials**	20](#_Toc215649801)

[**12. HIPAA Research Authorization**	23](#_Toc215649802)

[**13. Reasonably Anticipated Benefits**	24](#_Toc215649803)

[**14. Risks, Harms, & Discomforts**	24](#_Toc215649804)

[**15. Monitoring**	26](#_Toc215649805)

[**16. Data Analysis**	27](#_Toc215649806)

[**17. Bibliography**	28](#_Toc215649807)

# **1. Abbreviations and Definitions**

**Overview:** Cannabis (Cannabis sativa) contains dozens of cannabinoids. The most well-known cannabinoids are delta-9-tetrahydrocannabinol (THC) and cannabidiol (CBD). The term “medical cannabis” generally refers to the use of cannabis and cannabis-based products outside of the THC-based FDA approved products (e.g., nabilone, dronabinol). The types of available products through medical cannabis programs vary by state, with many providing capsules, oil, and vaporizing liquid. **Δ9-Tetrahydrocannabinol (THC):** THC is the primary psychoactive constituent of cannabis and acts as a partial agonist at both CB1 and CB2 receptors. Effects are observed clinically as impairments of learning, memory, spatial orientation, and attention during acute cannabis intoxication. THC can cause self-limited tachycardia, hypotension, orthostasis, xerostomia, and xerophthalmia. **Cannabidiol (CBD):** CBD lacks the THC-induced intoxicating properties that one traditionally thinks of as the “high” from cannabis use. Cannabidiol has observed anticonvulsant, anxiolytic, anti-inflammatory, and neuroprotective properties.

# **2. Background and Rationale**

Summarize and synthesize the available research, including published data, to provide justification for the study.

**Background**

**Sleep disturbance and reduced health-related quality of life (HRQOL) are pervasive and part of a vicious cycle leading to unmet needs among cancer patients.** Sleep disturbance occurs in 60% of cancer patients,^1^ three times higher than in the general population.^2^ Sleep disturbance is defined as difficulty initiating or maintaining sleep, waking up earlier than desired and being unable to fall back to sleep, and excessive daytime sleepiness. In adults with cancer, sleep disturbance may be caused by multiple, often co-occurring factors including diagnosis, type, and stage of cancer, treatment regimen, physical complaints (e.g., pain), and psychological distress.^3,4^ Sleep disturbance manifests as a vicious cycle with other symptoms such that significant symptom burden can contribute to sleep disturbance, and sleep disturbance may further compound symptom burden. Sleep disturbance is associated with numerous negative outcomes including reduced HRQOL, lower physical functioning, emotional distress, cognitive impairment, reduced immunity, and lower cancer survival.^2,5-7^ Medications for symptom management can be sedating and there is concern for polypharmacy with adding additional medications with sedating properties for sleep disturbance.^29,30^ Thus, many palliative providers address physical and psychological symptoms in addition to implementing non-pharmacologic and behavioral strategies as a first approach to management.^31^ While non-pharmacologic interventions have been shown to be beneficial, many of the strategies need time and participation/effort that may not be feasible in this population.^32^ Novel, safe approaches to further address sleep disturbance in cancer are critically needed.

HRQOL is an individual’s perceived physical and mental health that can be influenced by their clinical status and demographic background.^33,34^ Prior research,^35-37^ including from CO-I Krok-Schoen,^38^ found that the physical and mental HRQOL of cancer survivors was lower than that of cancer-free adults. This is alarming because lower HRQOL is associated with a myriad of longitudinal outcomes among adult patients, including higher symptom burden and comorbidities.^38-45^ Previous research demonstrated that 41-65% of cancer patients report a significant decline in HRQOL within the first year of a cancer diagnosis.^46-49^ Determinants to HRQOL are long-lasting; studies found that 5-15 years after cancer diagnosis HRQOL remains significantly lower among 30-40% of post-treatment cancer survivors.^50-52^ The interplay between sleep disturbance and HRQOL is insufficiently addressed in oncology, leading to continued unmet needs for both. It is essential to identify novel strategies by which improving sleep outcomes may additionally improve HRQOL.

**Cannabis products may be effective at improving sleep outcomes and HRQOL*.*** Use of cannabis products among adults has skyrocketed in the past two decades,^8^ with past-month cannabis use having increased 186% from 2007 (5.7%) to 2023 (16.3%) in this population.^9-11^ Although it is clear more research is needed to identify the potential benefits and harms of cannabis in general and among cancer patients in particular,^53-55^ it is possible cannabis may palliate symptoms and/or improve HRQOL in this population. Further, an American Society of Clinical Oncology report found that 60% of cancer patients wanted more information on the benefits of medical marijuana to alleviate their cancer-related symptoms.^56^

In trials among general populations with sleep disturbance, cannabis and cannabinoids improved sleep quality and duration and were well-tolerated.^57^ A 2022 systematic review noted that high-quality evidence of cannabis use for sleep in the general population remains limited due to heterogeneity of cannabis types, timing of administration, and measures of sleep outcomes. However, only mild AEs (headaches, sedation, dizziness) occurred more frequency at THC dosages >20mg/day and no serious AEs were reported.^58^ Randomized controlled trials examining the effect of cannabis extracts on sleep disturbance *in cancer patients* are few and vary in cannabis dosage and cannabinoid content, administration, symptom burden, and cancer diagnoses.^22-28^ Importantly, no trial was performed specifically among cancer patients with sleep disturbance; rather they were among patients experiencing other symptoms (e.g., pain, low appetite) regardless of their sleep.^22-28^ Most trials reported significant improvements in sleep disruption and increased quality of sleep.^22,23,26,27^ The remaining studies report no difference in sleep disturbance and quality between intervention and control groups,^24,28^ and none report worse sleep disturbance among cancer patients. Safety of cannabis use among cancer patients was consistent, with one study reporting more frequent minor AEs with higher THC doses.^22^

Experimental data on the effect of pharmaceutical cannabis or cannabinoids on HRQOL are limited. Several trials have examined HRQOL outcomes among patients with inflammatory bowel disease, epilepsy, and multiple sclerosis. Among them, small improvements were observed.^59,60^ To our knowledge, four trials have examined pharmaceutical cannabis for HRQOL among cancer patients and their findings are mixed.^61^ The proposed study will be the first to preliminarily examine the effect of a novel approach to address sleep disturbance and HRQOL specifically in patients with poor sleep quality.

**THC and CBD may promote sleep by interacting with the endocannabinoid system.** The endocannabinoid system is a neuromodulatory system that plays an important role in the central and peripheral nervous systems for maintaining homeostasis.^62,63^ Endocannabinoids are endogenously-produced, lipid-based neurotransmitters that bind to cannabinoid receptors CB1 and CB2.^62-64^ CB1 is primarily located in the nervous system, whereas CB2 is located primarily in immune cells and are involved in the inflammatory response.^64,65^ THC is a partial CB1 and CB2 receptor agonist, and its resulting activation leads to the modulation of peripheral sensory neurons (i.e., nociceptors) resulting in analgesic and euphoric effects.^66,67^ However, activation of CB1 in the amygdala at higher doses can result in unwanted neurological effects (e.g., paranoia).^68,69^ In contrast, CBD binds weakly to CB1 and CB2 and acts as an inverse agonist,^63^ resulting in anti-inflammatory and analgesic properties.^63,67^ Although the mechanism by which THC may aid sleep is incompletely understood, research suggests that it increases sleepiness and decreases sleep latency, and that lower THC:CBD ratios promote sedation through inhibition of the steroid hormone cortisol.^18,19^ A recent trial of a 1:1.5 THC:CBD oral solution versus a placebo in adults diagnosed with insomnia showed that cannabis administration resulted in a 30% increase in nighttime melatonin levels,^19,57^ a hormone that regulates the sleep-wake cycle. Administration of a standard unit (5mg)^70^ oral solution of THC in combination with CBD at a lower THC:CBD ratio may therefore increase sleepiness, decrease time to sleep, and promote sedation.

**Innovative measures for real-time assessment of sleep and symptom burden.** Ecological momentary assessment (EMA): One strategy employed by this team to measure the symptom trajectory among cancer patients is through EMA.^71^ This methodology may be particularly important because real-time, real-world changes in symptom burden can be assessed.^72,73^ EMA highlights individual differences in patient-reported outcomes and/or behavior, their distribution over time, factors affecting outcomes and behavior, and mutual associations between these factors.^73^ EMA complements traditional retrospective self-reports by providing real-time context to symptom burden and management strategies, and can reduce measurement error.^74^ EMA is useful and feasible in oncology research,^72^ cost-effective,^75^ and used successfully to examine sleep disturbance, HRQOL, and other symptoms in clinical populations.^71,76-80^ Actigraphy: Utilization of wearable devices to measure sleep is objective and minimizes measurement error. Actigraphy, validated against the gold-standard polysomnography (a “sleep study”) and self-reported indices, is a more convenient and cost-effective measure for long-term sleep assessment in patients’ homes.^81-83^ Actigraphy provides data points that the participant may not accurately recall, including sleep latency, total sleep time, sleep efficiency (percentage of time spent asleep while in bed), restlessness during sleep, and the number of instances of wakefulness after sleep onset. Actigraphy has been successfully used in clinical sleep studies in the oncology setting.^57,82,83^

**Rationale**

This pilot randomized, parallel-arm trial will examine the safety, feasibility, and preliminary efficacy of a cannabis gummy versus usual care in 50 new patients to the Ohio State University Comprehensive Cancer Center’s (OSUCCC) Palliative Care clinics who report sleep disturbance.

# **3. Objectives**

Describe the purpose of the study.

Our objective is to generate safety, feasibility, and preliminary efficacy data to support an R01 application for a large-scale clinical trial of cannabis for sleep disturbance and symptom palliation. To meet this objective, we will conduct a 5-week pilot randomized trial of a cannabis gummy (5mg THC and 5mg CBD) versus usual care in 50 cancer patients, after accounting for 5 dropouts per arm, reporting sleep disturbance at the OSUCCC’s Palliative Care clinics. Adverse events (AE) will be measured throughout the study. Sleep, HRQOL and other symptoms will be measured via self-report, including ecological momentary assessment (EMA), and actigraphy.

**Primary Objectives and Outcome Measures**

Describe the study objectives and outcomes.

| OBJECTIVES | ENDPOINTS | JUSTIFICATION FOR ENDPOINTS |
| --- | --- | --- |
| Primary | | |
| To determine the safety and feasibility of a nightly cannabis gummy versus usual care among palliative care patients at the OSUCCC. | Endpoints will be evaluated based on safety and feasibility (i.e., adherence and retention):  Safety will be evaluated by assessing the frequency, grade, and type of AE reported by participants. The frequency and percentage of participants experiencing each grade of AE will be reported for both groups.  Adherence will be measured as the proportion of participants who adhere to the study protocol, which includes consistent use of actigraphy, completion of self-reported measures, and measurement of self-reported cross-over events.  Retention will be the proportion of participants who complete all study assessments by Day 38. | These endpoints are necessary to determine whether a future trial can be conducted safely and effectively. |
|  |  |  |
| Secondary/Exploratory | | |
|  |  |  |
| To examine the preliminary efficacy of a nightly cannabis gummy versus usual care on sleep disturbance and HRQOL among palliative care patients at the OSUCCC. | Changes in these outcomes from baseline to Day 31 will provide initial insights into the intervention’s potential efficacy. Preliminary analyses will examine the effect of cannabis on sleep disturbance and HRQOL using means, standard deviations, and confidence intervals between follow-up measures. Secondary endpoints (e.g., actigraphy, EMA) will be assessed to explore cannabis’ broader effects. | Though exploratory, this endpoint will help determine the potential magnitude of effects to determine proper sample sizes in future trials. |

# **4. Study Design & Procedures**

|  | Y | N | Comments |
| --- | --- | --- | --- |
| 1. Is this a multi-site study? | ☐ | ☒ | If yes, complete the Multi-Site Research appendix. |
| 2. Does the research involve the use of an approved drug or biologic, use of an unapproved drug or biologic, or use a food or dietary supplement intended to diagnose, cure, treat, or mitigate a disease or condition? | ☒ | ☐ | If yes, complete the Drug appendix. |
| 3. Does the research involve the use of a device to evaluate its safety or effectiveness or use a humanitarian use device (HUD)? | ☐ | ☒ | If yes, complete the Device appendix. |

Provide information about all research interventions and activities that are to be performed.

This pilot randomized, parallel-arm trial will examine the safety, feasibility, and preliminary efficacy of a cannabis gummy versus usual care in 50 new cancer patients to the OSUCCC’s Palliative Care clinics who report sleep disturbance and have not used cannabis in the past 6 months. Detailed screening procedures will be conducted with participants including biometric screening. Participants will answer a baseline questionnaire focused on their cancer symptoms (e.g., sleep disturbance, HRQOL, pain, anxiety, nausea, and cognitive function) and will be provided with an ActiGraph device to be worn throughout the duration of the study. Participants assigned to the intervention group will receive a blood pressure monitor. At baseline, 50 participants (anticipating 5 dropouts per arm) will be randomized to either a nightly dose of a cannabis-based gummy for 30 days or usual care. Weblinks to brief EMA assessments will be sent via text message/email three days/week to record AEs, dose of intervention taken (among those randomized to intervention), blood pressure, crossover events (i.e., use of sleep aids or cannabis, depending on trial assignment), sleep quality, and HRQOL, phone calls may be used if requested by participants or in the event of technology challenges. On Day 31, patients will complete a comprehensive follow-up questionnaire to assess AEs, symptomology, and medication use as well as biometrics. One week following the end of the intervention period, patients will be sent a brief closeout questionnaire.

**Overview of study participation:**

**Initial screening** (Day -14):

New patients in the OSUCCC’s Palliative Care clinics who report sleep disturbance (≥4) in the Edmonton Symptom Assessment System (ESAS), the clinic’s symptom screener administered at every visit—will be identified by Palliative Care Clinic participating providers. Palliative Care Clinic providers will refer them to Dr. Stevens (CO-I) for preliminary eligibility review. Research staff will perform a chart review to confirm eligibility criteria prior to the phone screening.

**Phone screening** (Day -13):

Research staff will contact potentially eligible participants 24 hours following their in-clinic appointment to introduce the study and complete an eligibility screener. This screener will include the first item of the Patient-Reported Outcomes Measurement Information System (PROMIS^®^) Sleep Disturbance-Short Form 8a questionnaire. Patients who answer that their sleep was “poor” or “very poor” in the past week will be eligible. Eligibility screener will also include assessment of cannabis use, illicit medications, sleep medications, contaminant medications and others (see Inclusion and Exclusion Section). Eligible participants who express interest in participating will be scheduled for an Orientation & Enrollment visit and be asked to avoid taking sleep aids until randomization.

**Orientation, Biometric Screening & Enrollment** (Day -7):

Participants will meet with research staff at OSUCCC to discuss the study, ask questions, and if still interested, sign informed consent forms. Additionally, research staff will explain the actigraphy device and blood pressure monitor and give a brief tutorial on their use. Detailed dosing, storage, locking, and administration instruction for the cannabis gummy will be provided as well. Participants will be given phone numbers and instructions on how to contact a medical provider should an urgent adverse event occur.

Biometric screening procedures:

1. Vitals (blood pressure and heart rate)
2. Electrocardiograms [ECGs]: To perform the test, research staff will place electrodes on participant’s skin to record heart’s activity and look for abnormalities. Results will be sent to study physician for review.
3. Blood draw: Participants will be taken to CTR lab space and have research nurse take half a teaspoon (3ml) of blood for a comprehensive metabolic panel. At the end of the study visit, lab staff will take blood to OSU lab for analysis.
4. Orthostatic blood pressure

**Randomization** (Day 0):

Participants will be randomized to either a nightly dose of a cannabis-based gummy for 30 days or usual care based on a confirmation of their eligibility from the biometric screening. Participants will be randomized in sex and race strata and the unblinded randomization will be conducted using the Research Electronic Data Capture (REDCap) platform by study biostatistician who will have access to the allocation sequence. Participants randomized to the usual care arm will continue to receive routine care at the OSUCCC Palliative Care clinics. Routine care may include prescriptions to alleviate pain or anxiety, which may also treat sleep disturbance. Participants may additionally be prescribed non-habit-forming sleep aids (e.g., including melatonin and antidepressants) at the discretion of their provider (habit-forming sleep aids are rarely prescribed).

**Deliver study materials and complete baseline questionnaire** (Day 1):

Participants will answer a baseline questionnaire focused on their cancer symptoms, demographics, and ideation of self-harm; this questionnaire will be completed electronically or over the phone. Research staff will also meet with participants to provide them with the activity device, blood pressure monitor, and study intervention if randomized to the study drug. Participants on the intervention will begin their cannabis dosing one hour before bed in the evening of Day 1.

**Dosing and adverse events phone calls** (Days 2-5):

Research staff will call participants on the cannabis intervention arm days 2 through 5 to assess the participant’s first doses, see schedule blow. If the Day 1 dose was tolerable, patients will be asked to administer the same dose on the evening of Days 2 and 3. On Day 4, patients will be asked to administer the full dose (one 5mg THC/5mg CBD gummy). On day 5, participants will be called and assessed to determine whether they should remain at 100% of the target dose or be withdrawn from the study. If participant is unable to take the full 5mg dose at the end of day 5, they will be withdrawn from the study. Research staff will schedule the day 14 visit with participants at this time. The total duration of the intervention is 30 days.

**During the study** (Days 1-30):

Weblinks to brief EMA assessments will be sent via email or text message three days/week to record adverse events, dose of intervention taken, sleep quality, blood pressure, heart rate, and symptoms. Phone calls may be used if requested by participants or in the event of technology challenges.

**Mid-study visit** (day 14):

Research staff will conduct a video call with participants on the cannabis intervention arm on day 14 to assess safety. Research staff will ask the participants about any adverse events they might have experienced, any side effects they have experienced, and assess ideation of self-harm and other safety measures. Participants will complete vitals with the study staff. Research staff will also assess adherence with study procedures at this time.

**Follow-up assessment** (Day 31):

Participants will complete a follow-up electronic questionnaire to symptoms, ideation of self-harm, and medication use administered via web or phone. Participants will also return activity device, blood pressure monitor and remaining cannabis product. Follow up biometrics will include a comprehensive metabolic panel via blood draw, orthostatic hypotension and vitals. If orthostatic hypotension values are flagged or the patient is symptomatic, we will contact the study physician for review.

Biometric screening procedures:

1. Vitals (blood pressure and heart rate)
2. Blood draw: Participants will be taken to CTR lab space and have research nurse take half a teaspoon (3ml) of blood for a comprehensive metabolic panel. At the end of the study visit, lab staff will take blood to OSU lab for analysis.
3. Orthostatic blood pressure

**Closeout assessment** (Day 38):

One week following the end of the intervention period, patients will complete a brief electronic closeout questionnaire via phone or electronic device. The final questionnaire will assess their overall impressions of cannabis use (intervention arm only), sleep disturbance (PROMIS Sleep Disturbance Short-Form 8a), presence of rebounding or withdrawal symptoms, and post-trial use of cannabis or sleep aids. Finally, participants will receive compensation via ClinCard.

**Research Design**

Identify the research design appropriate to answer the question(s) under study.

This pilot randomized, parallel-arm trial will examine the safety, feasibility, and preliminary efficacy of a cannabis gummy versus usual care in 50 new cancer patients to the OSUCCC’s Palliative Care clinics who report sleep disturbance and have not used cannabis in the past 6 months.

**Detailed Study Procedures**

|  | Y | N | Comments |
| --- | --- | --- | --- |
| 1. Are you requesting expedited IRB review?   Note: The federal regulations establish two main criteria for an expedited review:   a. The research may not involve more than "minimal risk" (refer to appendix). b. The entire research project must be consistent with one or more of the federally defined categories (refer to appendix). | ☐ | ☒ | If yes, complete the Expedited Review appendix. |
| 2. Does the research involve genetic testing? | ☐ | ☒ | If yes, complete the Genetic Testing appendix. |

Describe the selected procedures sufficiently to justify their use in answering the defined research question(s).

This sample size, study design, and procedures are designed to provide sufficient data to assess the safety, feasibility, and preliminary efficacy of a cannabis gummy for treating sleep disturbance in cancer patients. The data collected will be used to refine the intervention and inform the design and power calculations for future trials.

Describe procedures for data collection. Be sure to specify the source records that will be used to collect data about participants. Upload all surveys, scripts, and data collection forms/spreadsheets to the Other Attachments section of the SmartForm.

Data collection will be collected and stored within institutional guidelines to maintain privacy and confidentiality. Our Advarra study database will be used as the source of data collection for both baseline and follow up questionnaires and the EMA questionnaires. Advarra has a HIPAA-compliant platform-level security plan supervised by OSUWMC IS, storage of a patient’s data in the platform poses no greater risk than entry of his or her data into other HIPAA-compliant OSUWMC information systems.

Describe the timeline for participant evaluations and the duration of project participation, for both individual study activities and total study participation, when applicable.

Participants will be enrolled in the study for 38 days.

| **Procedure** | **Initial Screening** | **Phone Screening** | **Enrollment** | **Orientation** | **Randomization** | **Baseline Questionnaire** | **Deliver study materials** | **Trial** | **Study Call Day 2-5** | **Mid-study visit** | **EMA assessment (3 times/week)** | **Follow up assessment** | **Closeout assessment** |
| --- | --- | --- | --- | --- | --- | --- | --- | --- | --- | --- | --- | --- | --- |
| **DAY** | **-14** | **-13** | **-7** | **-7** | **0** | **1** | **1** | **1-30** | **2-5** | **14** | **1-30** | **31** | **38** |
|  |  |  |  |  |  |  |  |  |  |  |  |  |  |
| Edmonton Symptom Assessment System | x |  |  |  |  |  |  |  |  |  |  |  |  |
| PROMIS Sleep Disturbance-Short Form 8a |  | x |  |  |  |  |  |  |  |  |  | x | x |
| Informed consent |  |  | x |  |  |  |  |  |  |  |  |  |  |
| Demographics |  |  |  |  |  | x |  |  |  |  |  |  |  |
| Medical History |  |  |  |  |  | x |  |  |  |  |  |  |  |
| Randomization |  |  |  |  | x |  |  |  |  |  |  |  |  |
| Pregnancy Test |  |  |  | x |  |  |  |  |  |  |  |  |  |
| Comprehensive metabolic panel |  |  |  | x |  |  |  |  |  |  |  | x |  |
| ECG |  |  |  | x |  |  |  |  |  |  |  |  |  |
| Orthostatic BP |  |  |  | x |  |  |  |  |  |  |  | x |  |
| Blood Pressure and HR |  |  |  | x |  |  |  |  |  | x | x | x |  |
| Dispense study drug |  |  |  |  |  |  | x |  |  |  |  |  |  |
| Administer study intervention |  |  |  |  |  |  |  | x |  |  |  |  |  |
| Concomitant medication review | x |  |  |  |  |  |  |  | x |  | x | x | x |
| Evaluation of suicidal ideation and behavior |  |  |  | x |  |  |  |  |  | x |  | x |  |
| PROMIS Sleep Disturbance 8a |  |  |  |  |  | x |  |  |  |  | x | x |  |
| PROMIS Sleep Impairment 8a |  |  |  |  |  | x |  |  |  |  |  | x |  |
| PROMIS-10 Global Health |  |  |  |  |  | x |  |  |  |  | x | x |  |
| PROMIS Pain Intensity 3a |  |  |  |  |  | x |  |  |  |  | x | x |  |
| PROMIS Pain Interference 8a |  |  |  |  |  | x |  |  |  |  |  | x |  |
| PROMIS Anxiety 8a |  |  |  |  |  | x |  |  |  |  | x | x |  |
| PROMIS Emotional Distress-Depression 8b |  |  |  |  |  | x |  |  |  |  | x | x |  |
| PROMIS Gastrointestinal Nausea and Vomiting 4a |  |  |  |  |  | x |  |  |  |  |  | x |  |
| PROMIS Cognitive Function 8a |  |  |  |  |  | x |  |  |  |  |  | x |  |
| Sleep actigraphy:   Sleep Latency, Total Sleep Time, Wake After Sleep Onset, Sleep Efficiency, Circadian Rhythm |  |  |  |  |  |  |  | x |  |  |  |  |  |
| Adverse event review and evaluation |  |  | x |  |  |  |  | x | x |  | x | x |  |
| Incentive payment |  |  |  |  |  |  |  |  |  |  |  |  | x |

Describe plans for long-term follow-up once all research related procedures are complete, if any, and specify what data will be collected during this period.

There are no plans for long-term follow-up.

**Alternatives to Participation**

Indicate any alternatives to participating in the research, if applicable, including available procedures or treatments outside the research that may be advantageous to the participant.

There are no alternatives to participating in the research. Patients could still pursue standard of care sleep care if not involved in this study.

# **5.** **Institutional Approvals**

1. Check all institutional approvals that apply to research being conducted at Ohio State.

☒ Comprehensive Cancer Center (CCC) Clinical Scientific Review Committee (CSRC): Approval or exemption required prior to IRB review for all cancer-related research.

Upload a copy of the approval letter to the Other Attachments section of the SmartForm.

☐ Institutional Biosafety Committee (IBC):

Approval required prior to IRB review for research involving biohazards (recombinant DNA, infectious or select agents, viruses, toxins), gene transfer, or xenotransplantation.

Upload a copy of the approval letter to the Other Attachments section of the SmartForm.

☐ Human Subject Radiation Committee (HSRC):

Approval required for research involving radiologic procedures for research purposes (e.g., non-clinical care X-rays, DEXA or CT scans, nuclear medicine procedures).

Complete the Radiation appendix and upload a copy of the HSRC approval letter to the Other Attachments section of the SmartForm.

# **6. Participant Population**

1. Specify the participant population(s). Check all participant groups that apply. For any population other than adults, complete the applicable appendix.

☒ Adults

☐ Adults with impaired decision-making capacity

☐ Children

☐ Neonates of uncertain viability

☐ Nonviable neonates

☐ Non-English-speaking individuals

☐ Pregnant women/fetuses (only if pregnant women will be intentionally recruited and/or	studied)

☐ Prisoners

Describe the sample population from which the study team plans to either recruit or access private, identifiable information for the research.

Cancer patients visiting the OSUCCC’s Palliative Care clinics.

Create a numbered list of the eligibility criteria that define who will be included in the final study sample (e.g., age, gender, condition).

**Inclusion Criteria**

1. New patients in the OSUCCC’s Palliative Care clinics who have histologically or cytologically confirmed malignancy of any anatomic site. This applies to either newly diagnosed cancer or those with preexisting malignancy receiving treatment.
2. Reported sleep disturbance (≥4) in the Edmonton Symptom Assessment System, the clinic’s symptom screener administered at every visit.
3. Patients with active cancers and are under any line of treatment or have received cancer treatment in the past 6 months (please see notable exceptions in the exclusion criteria – patients taking checkpoint inhibitors and investigational agents will not be eligible)
4. If patients are currently receiving cancer therapy, they must have been taking their current anti-cancer intervention/ therapy regimen for at least one month prior to enrollment (to ensure no safety or toxicity issues with study drug initiation).
5. Age ≥18 years.
6. English speaking with the ability to understand and the willingness to sign a written informed consent document.
7. Eastern Cooperative Oncology Group performance status ≤3.
8. Adequate organ and marrow function within the past 3 months or predating their current cancer regimen as defined below:

- total bilirubin: < 3X institutional upper limit of normal (1.5 mg/dl)

- AST(SGOT)/ALT(SGPT): < 3X institutional upper limit of normal

AST: < 3X institutional upper limit of normal (10-39 U/L)

ALT: < 3X institutional upper limit of normal (10-52 U/L)

- glomerular filtration rate (GFR): ≥30 mL/min/1.73 m2 using Cockcroft-Gault

- normal EKG; or non-normal EKG with no significant arrhythmias or severe CHF. A normal EKG defined as sinus rhythm with no ST or T wave changes any clinically concerning EKGs will be reviewed by the study physician to determine participant eligibility into the study.

9. Patients are allowed to take any type of analgesic pain medication at baseline, but must be on a stable dose of pain medication for two weeks and not requiring rapid dose escalation.
10. Individuals able to become pregnant will agree to practice a highly effective form of birth control. Contraception including oral birth control pills, IUD, condoms, abstinence must be used alone or in combination for the duration of enrollment.
11. Agree to study requirements, as described in the consent form:
    a. no driving, use of heavy machinery, or caring for children for 12 hrs after dose
    b. wearing fitness tracker 24/7 for the duration of the study and are able to charge the device once per week.
    c. no cannabis (i.e., outside of provided study drug), or illegal drug use (e.g., methamphetamine, non-prescription opioids or fentanyl)
    d. calls so that study staff can monitor dosing
    e. completing weekly EMA questionnaires

**Exclusion Criteria**

1. History of hypersensitivity to cannabis, THC, or CBD
2. Current, regular use of cannabis/marijuana, THC-containing prescription medications (dronabinol/Marinol/Syndros, nabilone/Cesamet), prescription CBD (Epidiolex), or over-the-counter CBD oil within the past 6 months
3. Concurrent use of medications that are contraindicated with medical cannabis: valproate, clobazam, warfarin, fluoxetine, disulfiram, tamoxifen, Ibrance, Gleevec, amphetamines, buprenorphine, methadone, alprazolam, amiodarone, fluconazole, ketoconazole, itraconazole, clarithromycin, ritonavir, erythromycin. The study physician will review eligibility for participants taking medications that are moderate to strong inhibitors and inducers of enzymes CYP3A4 (epidiolex, marinol), CYP2C19 (epidiolex), and CYP2C9 (marinol).
4. The following medications if being used to treat sleep: benzodiazepines or nonbenzodiazepine medications, antidepressants, antihistamines, supplements (e.g., melatonin)
5. Cardiac conditions contraindicated for cannabis use, moderate/severe or unstable CHF, symptomatic valvular heart disease, and uncontrolled arrhythmias as reviewed by the study team physician. Investigators will consult with a cardio-oncologist if needed.
6. Abnormal screening ECG with corrected QT (QTc) intervals of ≥450 (males) and ≥460 (females), as reviewed by the study physician.
7. Baseline orthostatic hypotension drop of BP ≥20 mmHg, or in diastolic BP of ≥10mmHg or experience of lightheadedness or dizziness during the test
8. Based on a measurement at baseline, we will exclude participants with current hypertension defined as BP >165/100, abnormal ECG results or a resting heart rate defined as >110bpm.
9. Concomitant use of checkpoint inhibitors (e.g., anti-PD1, PDL1, CTLA4).
10. Current use of investigational agents or <3 months after the use of investigational agents
11. Diagnosis of sleep apnea
12. Allergy to any constituent/ingredient contained in the edible doses
13. Psychiatric illness/social situations that would limit adherence with study requirements (e.g., bipolar disorder, psychosis). Mild/moderate mood disorders treated by medications that are not expected to increase cannabis-induced sedation/impairment are acceptable with physician approval.
14. Any known or suspected history or family history of schizophrenia, bipolar disorder, or other psychotic illness
15. Pregnant or breastfeeding
16. Sole caretaker of minor children living in the household
17. History of seizure disorder, epilepsy (controlled or uncontrolled)
18. Current legal obligations (parole, probation, incarceration)
19. Current substance use disorder (including cannabis use disorder), or currently enrolled in substance use treatment
20. Self-reported cannabis and synthetic cannabinoid use in the past 6 months (medical or non-medical use is exclusionary)
21. Self-reported illicit drug use in past 60 days (ex: methamphetamine, heroin, illicit fentanyl) or self-reported daily alcohol use
22. No access to internet/data or devices needed to participate in video calls (day 14) and EMA assessments

If appropriate, describe why certain populations may be excluded (e.g., non-English speaking individuals for studies involving informed consent).

Due to the small size of the study and the research team’s resources, we are unable to provide materials in other languages for participant inclusion. The goal of this trial is to collect data and refine the study design for a future, larger trial that could include non-English speaking participants.

**Number of Participants**

| The number of participants is defined as the number of individuals who agree to participate (i.e., those who provide consent or whose records are accessed) even if all do not prove eligible or complete the study. The total number of research participants may be increased only with prior IRB approval. | |
| --- | --- |
| 1. Indicate the number of participants to be enrolled locally. | 50 |
| 2. For multi-site studies, indicate the total number of participants to be enrolled across all sites. | N/A |

Explain how this number was derived (e.g., meets statistical power, limited population). If applicable, explain how many individuals are expected to agree to participate and how many evaluable participants are needed to conduct the study (excluding screen failures).

This sample size is designed to provide sufficient data to assess the safety, feasibility, and preliminary efficacy of a cannabis for treating sleep disturbance in cancer patients. The data collected will be used to refine the intervention and inform the design and power calculations for future larger trials. We anticipate a total of 10 dropouts during the study (five per arm), yielding n=40 total participants completing the study.

**Participant Identification**

Describe how potential participants will be identified (e.g., advertising, individuals known to the investigators, record review).

Palliative Care Clinic providers will identify new patients with sleep disturbances and refer them to Dr. Stevens (CO-I) for preliminary eligibility review. Upon passing the preliminary review, Dr. Stevens will refer patients to Alison Newton (Brasky Lab Supervisor) who will contact them to introduce the study and complete the eligibility screener. There will be no external advertising of the study to potential participants.

Explain how the investigator(s) will gain access to this population, as applicable, and provide evidence that you will be able to recruit the necessary number of participants to complete the study.

We will recruit 50 eligible patients, anticipating 10 dropouts (five per group), from the OSUCCC’s Palliative Care clinics over the course of 12 months. On average, the Palliative Care providers see ~30 new patients/week and ~1,500 new patients/year. Clinic data suggests that 60% will report sleep disturbance. Efforts will be made to recruit a diverse sample of participants, with a target of 25% of the sample consisting of individuals from minoritized racial and ethnic groups. This research team has had success recruiting from cancer clinics at the James and are well suited to complete this project.

**Participant Recruitment and Selection**

Describe how potential participants will be screened or otherwise determined to be eligible.

Clinic providers will identify new patients with sleep disturbances (from ESAS in clinic screener) and refer them to Dr. Stevens (CO-I) for preliminary eligibility review if they express interest upon a brief introduction about the study. Upon passing the preliminary review, Dr. Stevens will refer patients to Alison Newton (Brasky Lab Supervisor) who will contact them to introduce the study and complete the eligibility screener. This screener will assess cannabis use in the past six months, sleep aid usage, and include the first item of the Patient-Reported Outcomes Measurement Information System (PROMIS ®) Sleep Disturbance-Short Form 8a questionnaire. Patients who answer that their sleep was “poor” or “very poor” in the past week will be eligible. Additionally, the screener will assess patient medications for contraindications to medical cannabis. See screener and eligibility criteria for details. The research team that will complete the eligibility screening process is part of Theodore Brasky’s (PI) existing team and there is no plan for referral pathways or staff turnover. As this is a small pilot study, we do not anticipate the need for backup personnel.

Describe the recruitment process, including the setting in which recruitment will take place.

Recruitment will occur via phone call with potential participants. If the patient passes the screener and is interested in participating, they will be invited to a study orientation to complete informed consent documentation and complete study onboarding and biometric screenings. This orientation visit will take place in a private room at the OSU Center for Tobacco Research (3650 Olentangy River Rd.).

Explain how the recruitment process respects potential participants' privacy.

Recruitment will be conducted over the phone in the privacy of the patient's home/location of their choosing.

# **7. Cost to Participants and Incentives to Participate**

**Potential Costs/Reimbursements**

Describe any potential costs participants or their insurer will incur as a result of study participation (e.g., parking, study drugs, diagnostic tests). Indicate here which costs, if any, will be reimbursed or covered by the study.

There are no costs to participants as a result of study participation however, if a participant suffers an injury during the study the cost for this treatment will be billed to their medical or hospital insurance. This study has no funds set aside for the payment of health care expenses for participants.

**Incentives**

Describe any compensation or other incentives (e.g., free services, cash payments, gift cards, classroom credit) that will be offered to potential participants. Include the amount and timing of all incentives or compensation, the form of compensation (e.g., cash, check, gift card), and how the compensation will be received (e.g., mailed, in person, online). This information must also be included in the consent form.

Participants will receive monetary compensation via ClinCard for their participation in the baseline study visit, as well as completion of study assessments and regular use of the ActiGraph device. Participants will be reimbursed for their travel to the CTR for baseline and follow-up visits as well. Total compensation will be up to $200 per patient.

|  | Day | $ |
| --- | --- | --- |
| Orientation | -7 | 30 |
| Baseline | 0 | 20 |
| EMA | 1-30 | 70 |
| Follow-up | 31 | 20 |
| Closeout | 38 | 10 |
| ActiGraph |  | 20 |
| Travel |  | 30 |

# **8. Compensation for Research-Related Injury**

If the research involves greater than minimal risk to participants, describe the available compensation in the event of research-related injury or explain why no compensation is available.

There is no available compensation in the event of research related injury due to the constraints of the project funding. Participants will be informed that they should notify their Palliative care team in the event of an injury, and any cost of treatment will be billed to their medical or hospital insurance. The Ohio State University has no funds set aside for the payment of health care expenses for this study.

# **9. Informed Consent Process**

1. Specify the consent process(es) to be used for the study. Check all processes that apply. For any waivers or alterations, complete the applicable appendix.

☐ Informed Consent with Written Signature (e.g., on paper)

☒ Informed Consent with Electronic Signature

Note: If the method used to obtain electronic signatures does not qualify as a legally valid signature, also check “Waiver of Consent Documentation.” See guidance provided by the [Office of Technology and Digital Innovation](https://u.osu.edu/esigforinformedconsent/).

☐ Waiver of Consent/Parental Permission Documentation

☐ Deception (i.e., procedure in which investigators deliberately mislead participants during research by withholding information or providing false information).

Note: If this is selected, also check “Waiver or Alteration of the Consent Process.”

☐ Waiver or Alteration of the Consent Process

☐ Waiver of the Parental Permission Process

Describe the consent process. Explain when and where consent will be obtained and how participants or their legally authorized representatives will be provided sufficient opportunity to consider participation.

Interested and eligible patients who have passed the screening will be invited to an orientation visit at the OSU Clinical Research Center to provide written informed consent and medical record authorization or electronic consent on the Advarra (study database). Participants will be provided with as much time as necessary to decide whether to sign the consent form. Study staff will discuss the consent form with the participant after the participant has read the consent form. Study staff will ask questions to assess the individual’s understanding of the consent form.

Specify who will provide consent or permission (i.e., participant, legally authorized representative, parent and/or guardian).

The participant.

Explain how the possibility of coercion or undue influence will be minimized in the consent process.

Staff will be trained, and supervisors will not tolerate staff coercion or any other type of improper conduct. Participants will be told they can stop participating at any time and will be offered a copy of the consent form for their records. Staff may also be observed periodically to ensure procedures are being followed.

# **10. Privacy of Participants**

Describe the steps that will be taken to protect participants’ privacy and make them feel at ease with the research situation in terms of the questions being asked and the procedures being performed.

Information will be collected in a private setting by phone to reduce likelihood of personal, medical, or sensitive information being overheard. Also, we will be de-identifying the data to increase privacy and decrease the risk of any breach of confidentiality. Patients are told that participation is completely voluntary, and their decision will not impact their relationship with OSU/their physician or their care.

# **11. Confidentiality and Management of Study Materials**

|  | Y | N | Comments |
| --- | --- | --- | --- |
| 1. Does the research involve obtaining and storing participants’ data and/or biospecimens for future, unspecified, research? | ☐ | ☒ | If yes, complete the Repositories appendix. |

Describe the steps that will be taken to secure study data.

Study participants’ confidentiality will be maintained at all times. Participants will be assigned a unique study identification number (study ID). All study data will be identified by study ID only. Any electronic information will only be accessible to authorized study personnel who have the necessary password(s). All computer systems will be password-protected against intrusion. Access to computer-stored information will require simultaneous knowledge of the data format, computer language, file name and passwords. Safety measures will be implemented to minimize the risk of breaching confidentiality. Only authorized study personnel will have access to data identifying individuals. Data from questionnaires, electronic medical records, and data analyses will be stored in password secured databases. Data provided to the biostatistician for analysis purposes will not include identifiers. Access to information linking identifiers with the unique IDs will be restricted to the study manager and to others on a very strict need to know basis. Any paper documents with identifying information (e.g., informed consent forms) will be kept in a locked cabinet (Olentangy River Rd, Room 410B) inaccessible to staff other than authorized personnel or imaged to an electronic format and stored behind a firewall on a backed-up, password-protected server. The Advarra study database (baseline and follow-up questionnaires) will not pose additional risks to subjects beyond those inherent in receiving medical care or enrolling in a research study. In particular, because the Advarra has a HIPAA-compliant platform-level security plan supervised by OSUWMC IS, storage of a participant’s data in the platform poses no greater risk than entry of his or her data into other HIPAA-compliant OSUWMC information systems. Advarra also has additional internal controls described above to prevent unauthorized access to the study database users not affiliated with this protocol. Because of these stringent controls, the probability of a data breach is likely lower for this platform than for most study databases currently used for research. Every effort will be made to ensure that the safety and confidentiality of each participant is maintained and that they do not incur any undue risk.

Describe any procedures that will be used for quality control of collected data.

**Informed consent** --- Study staff will review both the documentation of the consenting process as well as a percentage of the completed consent documents. This review will evaluate compliance with good clinical practice, accuracy, and completeness.

**Source documents and electronic data** --- Data will be initially captured on Advarra which is directly entered into the study database.

**Intervention Fidelity** — Consistent delivery of the study interventions will be monitored throughout the intervention phase of the study.

**Protocol Deviations** – The study team will review protocol deviations on an ongoing basis and will implement corrective actions when the quantity or nature of deviations are deemed to be at a level of concern.

Describe how data (and if applicable, biospecimens) will be handled study wide.

Data collection will be the responsibility of the clinical trial staff. The investigator will be responsible for ensuring the accuracy, completeness, legibility, and timeliness of the data reported.

Clinical data (including adverse events (AEs), concomitant medications, and expected adverse reactions data) and clinical laboratory data will be entered into Advarra 21 CFR Part 11-compliant data capture system. The data system includes password protection and internal quality checks, such as automatic range checks, to identify data that appears inconsistent, incomplete, or inaccurate.

Specify the identifiable information that will be included in the data or associated with the biospecimens.

Surveys and phone assessments in addition to medical records will capture private data related to patient identification, cancer diagnosis, treatment/treatment toxicities, lifestyle, and quality of life. We do not plan to use biospecimen data for analyses, only for inclusion and exclusion purposes.

Specify where and how data or biospecimens will be stored and for how long.

Study documents will be retained for a minimum of 7 years after the last subject has completed participation in the study per HIPAA guidelines, or 2 years after approval of a marketing application in an International Council on Harmonisation (ICH) region and until there are no pending or contemplated marketing applications in an ICH region or until at least 2 years have elapsed since the formal discontinuation of clinical development of the study intervention, per FDA guidelines, whichever is greater.

All study materials will be kept on the OSU Wexner Medical Centers’ secure computer network that is behind a firewall and password protected. Questionnaire responses will be directly entered by study participants or direct entered by study staff. The OSU Electronic Data Capture (EDC) core, housed within the Department of Research Informatics Technology with major support from the Center for Clinical and Translational Science, will be utilized as a central provider for this project’s EDC Advarra support. All applications and processes that use patient information are governed by HIPAA during daily operations. The study will follow all existing procedures for protecting confidentiality and anonymity.

Specify who will have access to the data or biospecimens.

Only members of the research team will have access to the data.

Specify who will be responsible for receipt or transmission/transport of the data or biospecimens and how will transmission/transport will occur.

This study will not collect biospecimens as a data element. A blood draw will occur at orientation to assess patient eligibility; lab staff will transport blood samples to the lab for analysis.

Indicate what will happen to identifiable data at the end of the study (e.g., kept identifiable, coded, deidentified). If identifiers will be maintained, provide the rationale supporting this request. If the data will be deidentified, describe the process.

Identifiers will be permanently removed from the data and destroyed (resulting in de-identified data).

If data or biospecimens will be shared broadly with outside groups (i.e. dbGaP), specify what will be shared, with whom, in what form (e.g., identifiable, coded, deidentified/anonymized).

There are no plans to share this data with outside groups however research staff will maintain the de-identified data and may consider future research projects which may require shared data.

If data or biospecimens will be shared broadly with outside groups or databases (i.e. dbGaP), specify whether participants will have the ability to opt-in or remove the data/biospecimens from these outside groups later if they choose. Generally, if the materials are identifiable, a participant should be able to withdraw them unless retention is required under federal regulation (e.g., FDA).

There are no plans to share this data with outside groups.

# **12. HIPAA Research Authorization**

1. Is individually identifiable Protected Health Information (PHI) subject to the [HIPAA Privacy Rule](https://go.osu.edu/hipaaprivacysummary) requirements to be accessed, used, or disclosed in the study?

☐ No

☒ Yes

Note: The PHI obtained as part of this research must not be reused or disclosed to any other person or entity other than those with IRB approval, except as required by law or for authorized oversight of the research project, without additional approval. IRB approval must be obtained for other research involving the use or disclosure of this PHI.

2. If PHI is accessed, used, or disclosed, specify how authorization requirements will be met (check all that apply). For any waivers or alterations, complete the applicable appendix.

☒ Written Authorization

☒ Partial Waiver (for identification and recruitment purposes only)

☐ Full Waiver (authorization will not be obtained)

☐ Alteration (written authorization will not be obtained or all required elements will not be included)

Explain how the requested PHI (i.e., HIPAA identifiers and associated health information) are the minimum necessary information to accomplish the research objectives.

Screening for eligibility and maintaining a list of people who have been screened and/or declined to eliminate burden on patients. This includes the recording of information (noted above) among those who decline participation in order to ensure that the recruitment of individuals into the study is free of systematic error. This approach captures only data required to ensure eligibility and not mistakenly recontacted after refusing to participate. These data will also help identify populations who may be disproportionately impacted.

# **13. Reasonably Anticipated Benefits**

**Potential Direct Benefits:**

List the potential benefits that participants may expect as a result of this research study. If there are no direct benefits to individual participants, indicate so.

Study patients are not expected to benefit from their participation in the research study. The degree of risk to which individual study volunteers are at risk of a serious adverse event as a consequence of their research participation is relatively low under close medical supervision with expert medical staff.

**Potential Societal Benefits:**

List the potential benefits that society and/or others may expect as a result of this study.

The potential and probable benefits to be derived by society, in general, and for cancer patients specifically appear to be considerable. Knowledge gained from this study may help on a societal level in future evaluation and management of metastatic cancer patients who may use cannabis. Overall, the risk/benefit ratio appears favorable, and the conduct of this research seems well justified.

# **14. Risks, Harms, & Discomforts**

**Potential Risks, Harms, and/or Discomforts**

Describe all reasonably expected risks, harms, and/or discomforts that may apply to the research. At a minimum, include the risk of breach of data confidentiality. Discuss severity and likelihood of occurrence. As applicable, include potential risks to an embryo or fetus if a woman is or may become pregnant. Consider the range of risks, including physical, psychological, social, legal, economic, and any other potential risk to the study population.

**Possible side effects of cannabis gummy**

Potential side effects from the use of cannabis include, but are not limited to, the following: dizziness, anxiety, confusion, sedation, low blood pressure, impairment of short term memory, euphoria, difficulty in completing complex tasks, suppression of the body’s immune system, may affect the production of sex hormones that lead to adverse effects, inability to concentrate, impaired motor skills, paranoia, psychotic symptoms, general apathy, depression and/or restlessness. Marijuana may exacerbate schizophrenia in persons predisposed to that disorder. Many medical authorities claim that use of medical marijuana, especially by persons aged <25 years, can result in long-term problems with attention, memory, learning, drug abuse, and schizophrenia. Neurologic disorders: ataxia, excessive and purposeless motor activity of the extremities (hyperkinesis), seizures, lethargy, and prolonged coma (which may be life-threatening). Behavioral problems: anxiety, panic, dysphoria, dysphoria or agitation, sleepiness, euphoria, irritability, other changes in behavior Cognitive effects: impairs attention, concentration, episodic memory. Neuropsychiatric effects: feeling “high” marked by a euphoric, pleasurable feeling and a decrease in anxiety, alertness, depression, and tension. Perceptual changes including the sensation that colors are brighter and music is more vivid. Mystical thinking, increased self-consciousness and depersonalization, paranoia and other signs of psychosis. Cardiovascular disorders: tachycardia, hypertension, chest pain, angina, myocardial infarction dyspnea, cardiac arrythmia, and stroke. Respiratory disorders: dyspnea, pseudopneumothorax, pseudomediastinum, cough, sputum production, and wheezing. Gastrointestinal disorders: nausea, vomiting, and diarrhea.

**Questionnaires**

Some participants may experience psychological discomfort stemming from thinking about their cancer diagnosis, symptoms, and symptom management, and it is possible that some questions on the questionnaire may provide minimal (or no) psychological stress. Participants will be advised that they are not required to answer any questions that they do not wish to answer and can skip them without penalty to them.

**Confidentiality**

There is a minimal risk of loss of confidentiality in this study. Precautions will be taken to protect computerized or electronic data from unauthorized disclosure, tampering, or damage by controlling access to the computers and files that hold this information. Access to computer files will only be obtained through passwords and will only be accessed by Institutional Review Board (IRB)-authorized research study personnel. Only authorized research study personnel will have permission to examine computer records to identify which patients are eligible to approach.

**Pregnancy related risks**

This study excludes women who are pregnant (as indicated by a positive urine pregnancy test) or nursing, and women who are of child-bearing potential and sexually active who are not practicing a highly effective means of birth control (i.e., implants, injectables, combined oral contraceptives, progestin containing IUDs, or vasectomized partner). In the unlikely event that one of the participants becomes pregnant during the trial, that person would be discontinued from further cannabis dosing as well as discontinued from any study procedures that would interfere with appropriate medical response and care.

**Unknown risks**

There may be side effects and discomforts of this study that are not yet known.

**Risks Associated with Study Procedures in This Study:**

• Electrocardiograms [ECGs]: To perform the test, electrodes, i.e., stickers with wires attached, will be placed on skin to record participant’s heart’s activity. No pain or other side effects are expected to be associated with these tests, but participants may feel some discomfort, similar to pulling off an adhesive bandage, when the technician removes the electrodes after the procedure.

• Blood Samples: Side effects of having blood taken may include pain, redness, swelling, and/or bruising where the needle enters the body. Rare instances of fainting, excess bleeding, blood clotting, or infection have occurred.

**Other risks**

There are risks of drug diversion and accidental ingestion.

**Risk Mitigation**

Describe how risks, harms, and/or discomforts will be minimized. Address all risks described in the section above.

Study staff will be trained. Only study investigators and staff who have completed their institutional or the NIH human subjects training will be able to work on this project. Documentation of completion will be maintained on file at OSU. Study subjects’ confidentiality will be maintained at all times. Every effort will be made to ensure that the safety and confidentiality of each participant is maintained and that they do not incur any undue risk. Participants have the right to withdraw from the research study at any time they so desire.

Storage of a patient’s data in the Advarra platform poses no greater risk than entry of his or her data into other HIPAA-compliant OSUWMC information systems. Advarra also has additional internal controls to prevent unauthorized access to the study database by users not affiliated with this protocol. Because of these stringent controls, the probability of a data breach is likely lower for this platform than for most study databases currently used for research. Every effort will be made to ensure that the safety and confidentiality of each patient is maintained and that they do not incur any undue risk.

To mitigate the risk of accidental ingestion and drug diversion our research team will use two different types of locking procedures to avoid this risk. First the cannabis will be locked in weekly pill containers that have individual number locks. Access codes will be provided to participants at the start of each week. The second locking mechanism is a waterproof bag with a lock on the zipper, again, the access code will be provided to the participant at the start of the study.

See Monitoring section for details on adverse event reporting and handling.

**Information about the cannabis gummy from the manufacturer:**

If testing will be performed to identify individuals who may be at increased risk (e.g., pregnant women, individuals with HIV/AIDS, depressive disorders), address the timing and method of testing; include how positive test results will be handled.

This study excludes women who are pregnant (as indicated by a positive urine pregnancy test at orientation) or nursing, and women who are of child-bearing potential and sexually active who are not practicing, unable to practice, or do not agree to practice a highly effective means of birth control (i.e., implants, injectables, combined oral contraceptives, progestin containing IUDs, or vasectomized partner) alone or in combination. In the unlikely event that one of the participants becomes pregnant during the trial, that person would be discontinued from further cannabis dosing as well as discontinued from any study procedures that would interfere with appropriate medical response and care.

# **15. Monitoring**

|  | Y | N | Comments |
| --- | --- | --- | --- |
| 1. Does the research involve greater than minimal risk (i.e., the harms or discomforts described are beyond those ordinarily encountered in daily life or during the performance of routine physical or psychological tests)? | ☒ | ☐ | If yes, describe a data and safety monitoring plan below. |

Describe the plan to oversee and monitor data collected to ensure participant safety and data integrity. If the plan is outlined in a separate document, upload it to the documents page of the application SmartForm. If not, describe it below and include the following:

- The information that will be evaluated (e.g., incidence and severity of actual harm compared to that expected)
- Who will perform the monitoring (e.g., investigator, sponsor, or independent monitoring committee/board)
- Timing of monitoring (e.g., at specific points in time, after a specific number of participants have been enrolled/treated) and
- Decisions to be made as a result of the monitoring process (e.g., provisions to stop the study early for unanticipated problems)

1. **Safety and Other Assessments**

The primary clinician will identify/record AEs (i.e., the emergence of any untoward physical or psychological events or symptoms) and safety concerns throughout the course of the study. The type, severity, and frequency of adverse events will be collected in order to identify and characterize any safety concerns that may arise.

2. **Adverse Events and Serious Adverse Events**
   1. Definition of Adverse Events (AE)

This protocol uses the definition of adverse event from 21 CFR 312.32 (a): any untoward medical occurrence associated with the use of an intervention in humans, ***whether or not considered intervention-related***. 

2. Definition of Serious Adverse Events (SAE)

In agreement with OSU Office of Responsible Research practices Policy #22b, we define a serious adverse event as one that is fatal or life threatening, permanently disabling, requires hospitalization, or results in significant disability, congenital anomaly or birth defect.

3. **Classification of an Adverse Event**

Severity of Event: For adverse events (AEs) not included in the protocol defined grading system, the following guidelines will be used to describe severity.

- **Mild** – Events require minimal or no treatment and do not interfere with the participant’s daily activities.
- **Moderate** – Events result in a low level of inconvenience or concern with the therapeutic measures. Moderate events may cause some interference with functioning.
- **Severe** – Events interrupt a participant’s usual daily activity and may require systemic drug therapy or other treatment. Severe events are usually potentially life-threatening or incapacitating. Of note, the term “severe” does not necessarily equate to “serious”.

4. **Relationship to Study Intervention**

All adverse events (AEs) will have their relationship to study procedures, including the intervention, assessed by an appropriately trained clinician based on temporal relationship and his/her clinical judgment. The degree of certainty about causality will be graded using the categories below.

- **Definitely Related** – There is clear evidence to suggest a causal relationship, and other possible contributing factors can be ruled out. The clinical event, including an abnormal laboratory test result, occurs in a plausible time relationship to study procedures administration and cannot be explained by concurrent disease or other drugs or chemicals. The response to withdrawal of the study procedures should be clinically plausible. The event must be pharmacologically or phenomenologically definitive.
- **Probably Related** – There is evidence to suggest a causal relationship, and the influence of other factors is unlikely. The clinical event, including an abnormal laboratory test result, occurs within a reasonable time after administration of the study procedures, is unlikely to be attributed to concurrent disease or other drugs or chemicals, and follows a clinically reasonable response on withdrawal.
- **Potentially Related** – There is some evidence to suggest a causal relationship (e.g., the event occurred within a reasonable time after administration of study procedures). However, other factors may have contributed to the event (e.g., the participant’s clinical condition, other concomitant events). Although an AE may rate only as “possibly related” soon after discovery, it can be flagged as requiring more information and later be upgraded to “probably related” or “definitely related”, as appropriate.
- **Unlikely to be related** – A clinical event, including an abnormal laboratory test result, whose temporal relationship to study procedures administration makes a causal relationship improbable (e.g., the event did not occur within a reasonable time after administration of the study procedures) and in which other drugs or chemicals or underlying disease provides plausible explanations (e.g., the participant’s clinical condition, other concomitant treatments).
- **Not Related** – The AE is completely independent of study procedures administration, and/or evidence exists that the event is definitely related to external causes separate from the study.

5. **Expectedness**

The principal investigator and medical monitor will be responsible for determining whether an AE is expected or unexpected. An AE will be considered unexpected if the nature, severity, or frequency of the event is not consistent with the risk information previously described for the study procedures.

6. **Time Period and Frequency for Event Assessment and Follow-Up**

The occurrence of an AE or SAE may come to the attention of study personnel during study phone calls of a study participant presenting for medical care, or upon review by a study monitor.

All AEs, not otherwise precluded per the protocol, will be captured on the appropriate case report form (CRF). Information to be collected includes event description, time of onset, clinician’s assessment of severity, relationship to study procedures (assessed only by those with the training and authority to make a diagnosis), and time of resolution/stabilization of the event. All AEs occurring while on study will be documented appropriately regardless of relationship. All AEs will be followed to adequate resolution.

Any medical or psychiatric condition that is present at the time that the participant is screened will be considered as baseline and not reported as an AE. However, if the study participant’s condition deteriorates at any time during the study, it will be recorded as an AE.

Changes in the severity of an AE will be documented to allow an assessment of the duration of the event at each level of severity to be performed. Documentation of onset and duration of each episode will be maintained for AEs characterized as intermittent.

A trained member of the study staff will record events with start dates occurring any time after informed consent is obtained until 7 (for non-serious AEs) or 30 days (for SAEs) after the last day of study participation. At each study contact, the investigator will inquire about the occurrence of AE/SAEs since the last contact. Events will be followed for outcome information until resolution or stabilization.

7. **Adverse Event Reporting**

As per OSU IRB guidelines (policy #22b, section 4), adverse events will be reported to the IRB using the Event Report application in Buck-IRB within 10 days of the investigators or research staff member’s learning of the event. Events resulting in temporary or permanent interruption of study activities by the investigator, sponsor, or Data and Safety Monitoring Board (DSMB) to avoid potential harm to subjects will be reported within 48 hours. Events that may represent unanticipated problems involving risks to subjects or others will be reported, regardless of whether they occur during or after the study or involve a subject who has withdrawn from or completed study participation. If changes to the research or consent process are proposed as a result of the event, or if additional information will be provided to current and/or past participants, an amendment request will be submitted for IRB review.

The data and safety monitoring plan will involve the continuous evaluation of safety, data quality and data timeliness. Investigators will conduct continuous review of data and patient safety at their study team meetings established by the principal investigator or study manager. The PI of the trial will review toxicities and responses of the trial where applicable at these meetings and determine if the risk/benefit ratio of the trial changes. Frequency and severity of adverse events will be reviewed by the PI and compared to what is known about the intervention/agent/device from other sources; including published literature, scientific meetings, and discussions with the sponsors to determine if the trial should be terminated before completion. Adverse events and the overall progress of the study will also be reviewed by the OSUCCC Data and Safety Monitoring Committee (DSMC). The PI will also submit a summary report, at least annually, that will be reviewed by the committee per the IRB of record as per the policies of the IRB. All Serious Adverse Events are to be submitted to the DSMC for their review per reporting requirements outlined in the protocol and will be reviewed by the DSMC on a monthly basis. All deaths on study will be reviewed within 2 business days. All adverse events will be submitted as part of the summary report.

8. **Serious Adverse Event Reporting**

In consultation with the PI, a trained member and medical monitor (Dr. Erin Stevens) of the study team will be responsible for conducting an evaluation of a serious adverse event and shall report the results of such evaluation to the reviewing IRB as soon as possible, but in no event later than 10 working days after the investigator first learns of the event.

9. **Reporting Events to Participants**

If any SAEs reported during the study are categorized as probably or definitely related to the cannabis intervention, we will modify our informed consent document to include mention of these events for future participants and update consent for any participants that are currently enrolled but have not yet completed the study.

10. **Participant death**

In the event of a participant death while on study, research staff will be notified by the study physician and will meet with next of kin at an OSU clinic or facility near to the participant’s home in order to collect remaining study drug and research devices.

# **16. Data Analysis**

**Internal/External Validity**

Describe measures that have been taken to avoid study bias (consider the threats to internal/external validity).

This pilot study will be conducted without blinding. The intervention group (cannabis gummy) and the control group (usual care) will be aware of their group assignments. A stratified random sampling will ensure trial arm equity by race (white/non-white) and sex and will be performed by study staff.

**Data Analysis Techniques**

Specify the analytic techniques the researcher will use to answer the study questions. Indicate the statistical procedures (e.g. specific descriptive or inferential tests) that will be used and why the procedures are appropriate. Note: The IRB does not need the actual formulas to be used, just a description of them. If applicable, specify the proposed analytic approaches for qualitative data.

**GENERAL APPROACH**

We will recruit 50 patients (25 per group) with an anticipated attrition rate of 20%. This sample size is designed to provide sufficient data to assess safety, feasibility, and preliminary efficacy of a cannabis gummy for treating sleep disturbance in cancer patients. The data collected will be used to refine the intervention and inform the design and power calculations for future trials. All outcome variables will first be examined with descriptive statistical analyses that focus on describing and understanding patterns and distributions within each of the study arms.

**ANALYSIS OF THE PRIMARY EFFICACY ENDPOINT(S)**

Safety (aim 1): Safety will be evaluated by assessing the frequency, grade, and type of AE reported by participants. The frequency and percentage of participants experiencing each grade of AE will be reported for both groups. Comparisons of AE incidence between the intervention and control groups will be conducted using Fisher’s exact test. Feasibility (aim 1): Feasibility will be evaluated based on adherence, crossover, and retention. Adherence will be measured as the proportion of participants who adhere to the study protocol, which includes consistent use of actigraphy and completion of self-reported measures. We anticipate that participants will achieve ≥80% adherence across measures (including <20% crossover). Retention will be the proportion of participants who complete all study assessments by Day 38, with an expected retention rate of 80%. Adherence rates will be analyzed using descriptive statistics. Logistic regression will be used to identify factors associated with adherence and retention.

Exploratory analyses (aim 2): Given the exploratory nature of this aim, formal hypothesis testing will not be conducted. Instead, the objective is to present changes in these outcomes from baseline to Day 31 to provide initial insights into the intervention’s potential efficacy. Preliminary analyses will examine the effect of cannabis on sleep disturbance and HRQOL using means, standard deviations, and confidence intervals between follow-up measures. Secondary endpoints (e.g., actigraphy, EMA) will be assessed to explore cannabis’ broader effects.

# **17. Bibliography**

1. Al Maqbali M, Al Sinani M, Alsayed A, Gleason AM. Prevalence of Sleep Disturbance in Patients With Cancer: A Systematic Review and Meta-Analysis. Clin Nurs Res. 2022;31(6):1107-1123.

2. Palesh OG, Roscoe JA, Mustian KM, et al. Prevalence, demographics, and psychological associations of sleep disruption in patients with cancer: University of Rochester Cancer Center-Community Clinical Oncology Program. J Clin Oncol. 2010;28(2):292-298.

3. Howell D, Oliver TK, Keller-Olaman S, et al. Sleep disturbance in adults with cancer: a systematic review of evidence for best practices in assessment and management for clinical practice. Ann Oncol. 2014;25(4):791-800.

4. Beverly Hery CM, Janse SA, Van Zee KJ, Naftalis EZ, Paskett ED, Naughton MJ. Factors associated with insomnia symptoms over three years among premenopausal women with breast cancer. Breast Cancer Res Treat. 2023;202(1):155-165.

5. Baker TA, Krok-Schoen JL, McMillan SC. Identifying factors of psychological distress on the experience of pain and symptom management among cancer patients. BMC Psychol. 2016;4(1):52.

6. Davidson JR, MacLean AW, Brundage MD, Schulze K. Sleep disturbance in cancer patients. Soc Sci Med. 2002;54(9):1309-1321.

7. Tong HH, Li JR, Feng Y, Li SW, Qiu H, Hong JF. The Association Between Sleep Disturbance and Proinflammatory Markers in Patients With Cancer: A Meta-analysis. Cancer Nurs. 2023;46(2):E91-e98.

8. Maxwell CJ, Jesdale BM, Lapane KL. Recent Trends in Cannabis Use in Older Americans. Ann Intern Med. 2021;174(1):133-135.

9. Solomon HV, Greenstein AP, DeLisi LE. Cannabis Use in Older Adults: A Perspective. Harv Rev Psychiatry. 2021;29(3):225-233.

10. Han BH, Palamar JJ. Trends in Cannabis Use Among Older Adults in the United States, 2015-2018. JAMA Intern Med. 2020;180(4):609-611.

11. Substance Abuse and Mental Health Services Administration. Marijuana Use in Lifetime, Past Year, and Past Month: Among People Aged 12 or Older; by Detailed Age Category, Percentages, 2021 and 2022 (Table 1.7B). https://www.samhsa.gov/data/sites/default/files/reports/rpt42728/NSDUHDetailedTabs2022/NSDUHDet ailedTabs2022/NSDUHDetTabsSect1pe2022.htm. Published 2023. Accessed 12/21/2023, 2023.

12. Leas EC, Nobles AL, Caputi TL, Dredze M, Smith DM, Ayers JW. Trends in Internet Searches for Cannabidiol (CBD) in the United States. JAMA Netw Open. 2019;2(10):e1913853.

13. van den Beuken-van Everdingen MH, de Graeff A, Jongen JL, Dijkstra D, Mostovaya I, Vissers KC. Pharmacological Treatment of Pain in Cancer Patients: The Role of Adjuvant Analgesics, a Systematic Review. Pain Pract. 2017;17(3):409-419.

14. Schuermeyer J, Salomonsen-Sautel S, Price RK, et al. Temporal Trends in Marijuana Attitudes, Availability and Use in Colorado Compared to Non-Medical Marijuana States: 2003-11. Drug and Alcohol Dependence. 2014;140:145-155.

15. Victorson D, McMahon M, Horowitz B, Glickson S, Parker B, Mendoza-Temple L. Exploring Cancer Survivors' Attitudes, Perceptions, and Concerns about Using Medical Cannabis for Symptom and Side Effect Management: A Qualitative Focus Group Study. Complementary Therapies in Medicine. 2019;47:102204-102212.

16. Pacula RL, Smart R. Medical Marijuana and Marijuana Legalization. Annual Review of Clinical Psychology. 2017;13:397-419.

17. Steele G, Arneson T, Zylla D. A Comprehensive Review of Cannabis in Patients with Cancer: Availability in the USA, General Efficacy, and Safety. Current Oncology Reports. 2019;21(1):1-12.

18. Nicholson AN, Turner C, Stone BM, Robson PJ. Effect of Delta-9-tetrahydrocannabinol and cannabidiol on nocturnal sleep and early-morning behavior in young adults. J Clin Psychopharmacol. 2004;24(3):305-313.

19. Low ZXB, Lee XR, Soga T, Goh BH, Alex D, Kumari Y. Cannabinoids: Emerging sleep modulator. Biomed Pharmacother. 2023;165:115102.

20. Brasky TM, Newton AM, Conroy S, et al. Marijuana and cannabidiol use prevalence and symptom management among cancer patients. Cancer Res Commun. 2023;3(9):1917-1926.

21. Braun IM, Bohlke K, Abrams DI, et al. Cannabis and Cannabinoids in Adults With Cancer: ASCO Guideline. J Clin Oncol. 2024;42(13):1575-1593.

22. Portenoy RK, Ganae-Motan ED, Allende S, et al. Nabiximols for opioid-treated cancer patients with poorly-controlled chronic pain: a randomized, placebo-controlled, graded-dose trial. The Journal of Pain. 2012;13(5):438-449.

23. Lichtman AH, Lux EA, McQuade R, et al. Results of a Double-Blind, Randomized, Placebo-Controlled Study of Nabiximols Oromucosal Spray as an Adjunctive Therapy in Advanced Cancer Patients with Chronic Uncontrolled Pain. Journal of Pain and Symptom Management. 2018;55(2):179-188.e171.

24. Johnson JR, Burnell-Nugent M, Lossignol D, Ganae-Motan ED, Potts R, Fallon MT. Multicenter, double blind, randomized, placebo-controlled, parallel-group study of the efficacy, safety, and tolerability of THC: CBD extract and THC extract in patients with intractable cancer-related pain. Journal of pain and symptom management. 2010;39(2):167-179.

25. Fallon MT, Albert Lux E, McQuade R, et al. Sativex oromucosal spray as adjunctive therapy in advanced cancer patients with chronic pain unalleviated by optimized opioid therapy: two double-blind, randomized, placebo-controlled phase 3 studies. Br J Pain. 2017;11(3):119-133. 26. Brisbois TD, de Kock IH, Watanabe SM, et al. Delta-9-tetrahydrocannabinol may palliate altered chemosensory perception in cancer patients: results of a randomized, double-blind, placebo-controlled pilot trial. Ann Oncol. 2011;22(9):2086-2093.

27. Turcott JG, Del Rocío Guillen Núñez M, Flores-Estrada D, et al. The effect of nabilone on appetite, nutritional status, and quality of life in lung cancer patients: a randomized, double-blind clinical trial. Support Care Cancer. 2018;26(9):3029-3038.

28. Côté M, Trudel M, Wang C, Fortin A. Improving Quality of Life With Nabilone During Radiotherapy Treatments for Head and Neck Cancers: A Randomized Double-Blind Placebo-Controlled Trial. Ann Otol Rhinol Laryngol. 2016;125(4):317-324.

29. Sela RA, Watanabe S, Nekolaichuk CL. Sleep disturbances in palliative cancer patients attending a pain and symptom control clinic. Palliat Support Care. 2005;3(1):23-31.

30. Graci G. Pathogenesis and management of cancer-related insomnia. J Support Oncol. 2005;3(5):349- 359.

31. Jeon MS, Allcroft P, Brown LR, et al. Assessment and Management of Sleep Disturbance in Palliative Care Settings. J Palliat Med. 2024;27(7):905-911.

32. Howell D. Sleep Difficulties in Palliative Care Patients. In: MacLeod RD, Van den Block L, eds. Textbook of Palliative Care. Cham: Springer International Publishing; 2020:1-31.

33. Reeve BB, Potosky AL, Smith AW, et al. Impact of cancer on health-related quality of life of older Americans. J Natl Cancer Inst. 2009;101(12):860-868.

34. Cella DF. Measuring quality of life in palliative care. Semin Oncol. 1995;22(2 Suppl 3):73-81.

35. Mielck A, Vogelmann M, Leidl R. Health-related quality of life and socioeconomic status: inequalities among adults with a chronic disease. Health Qual Life Outcomes. 2014;12:58.

36. Inoue-Choi M, Lazovich D, Prizment AE, Robien K. Adherence to the World Cancer Research Fund/American Institute for Cancer Research recommendations for cancer prevention is associated with better health-related quality of life among elderly female cancer survivors. J Clin Oncol. 2013;31(14):1758-1766.

37. Baker F, Denniston M, Haffer SC, Liberatos P. Change in health-related quality of life of newly diagnosed cancer patients, cancer survivors, and controls. Cancer. 2009;115(13):3024-3033.

38. Krok-Schoen JL, Pisegna J, Arthur E, Ridgway E, Stephens C, Rosko AE. Prevalence of lifestyle behaviors and associations with health-related quality of life among older female cancer survivors. Support Care Cancer. 2021;29(6):3049-3059.

39. Pergolotti M, Deal AM, Williams GR, et al. Activities, function, and health-related quality of life (HRQOL) of older adults with cancer. J Geriatr Oncol. 2017;8(4):249-254.

40. Decoster L, Quinten C, Kenis C, et al. Health related quality of life in older patients with solid tumors and prognostic factors for decline. J Geriatr Oncol. 2019;10(6):895-903.

41. Klapheke AK, Keegan THM, Ruskin R, Cress RD. Pre-diagnosis health-related quality of life and survival in older women with endometrial cancer. Support Care Cancer. 2020;28(10):4901-4909.

42. Park J, Rodriguez JL, O'Brien KM, et al. Health-related quality of life outcomes among breast cancer survivors. Cancer. 2021;127(7):1114-1125.

43. Smith AW, Reeve BB, Bellizzi KM, et al. Cancer, comorbidities, and health-related quality of life of older adults. Health Care Financ Rev. 2008;29(4):41-56.

44. Utley M, Adeyanju T, Bernardo B, Paskett ED, Krok-Schoen JL. The association between mental health, social support and physical health outcomes among older female cancer survivors. J Geriatr Oncol. 2022;13(6):834-838.

45. Ximenes CRC, Bergmann A, Lima JTO, et al. Prospective Analysis of Health-Related Quality of Life in Older Adults With Cancer. J Patient Exp. 2020;7(6):1519-1525.

46. Farris MS, Kopciuk KA, Courneya KS, McGregor SE, Wang Q, Friedenreich CM. Identification and prediction of health-related quality of life trajectories after a prostate cancer diagnosis. Int J Cancer. 2017;140(7):1517-1527.

47. Dunn J, Ng SK, Breitbart W, et al. Health-related quality of life and life satisfaction in colorectal cancer survivors: trajectories of adjustment. Health Qual Life Outcomes. 2013;11:46. 48. Arthur EK, Nikahd M, Hyer JM, et al. Associations of Demographic and Social Factors on HealthRelated Quality-of-Life Changes Among Older Women With Breast or Gynecologic Cancer. Oncol Nurs Forum. 2024;51(2):127-141.

49. Park JH, Jung YS, Kim JY, Jo Y, Bae SH. Trajectories of health-related quality of life in breast cancer patients. Support Care Cancer. 2020;28(7):3381-3389.

50. Cheng Z, Johar A, Lagergren J, Schandl A, Lagergren P. Health-related quality of life trajectories up to 15 years after curative treatment for esophageal cancer: a prospective cohort study. Int J Surg. 2024;110(3):1537-1545.

51. Tran TXM, Jung SY, Lee EG, et al. Long-term trajectory of postoperative health-related quality of life in young breast cancer patients: a 15-year follow-up study. J Cancer Surviv. 2023;17(5):1416-1426.

52. Chambers SK, Ng SK, Baade P, et al. Trajectories of quality of life, life satisfaction, and psychological adjustment after prostate cancer. Psychooncology. 2017;26(10):1576-1585.

53. Whiting PF, Wolff RF, Deshpande S, et al. Cannabinoids for Medical Use: A Systematic Review and Meta-analysis. JAMA. 2015;313(24):2456-2473.

54. Pratt M, Stevens A, Thuku M, et al. Benefits and harms of medical cannabis: a scoping review of systematic reviews. Syst Rev. 2019;8(1):320.

55. van den Elsen GA, Ahmed AI, Lammers M, et al. Efficacy and safety of medical cannabinoids in older subjects: a systematic review. Ageing Res Rev. 2014;14:56-64.

56. Oncology ASoC. ASCO's National cancer opinion survey. Harris Poll on behalf of ASCO. Alexandria, VA: American Society of Clinical Oncology;2019.

57. Ried K, Tamanna T, Matthews S, Sali A. Medicinal cannabis improves sleep in adults with insomnia: a randomised double-blind placebo-controlled crossover study. J Sleep Res. 2023;32(3):e13793.

58. Velzeboer R, Malas A, Boerkoel P, et al. Cannabis dosing and administration for sleep: a systematic review. Sleep. 2022;45(11).

59. Goldenberg M, Reid MW, IsHak WW, Danovitch I. The impact of cannabis and cannabinoids for medical conditions on health-related quality of life: A systematic review and meta-analysis. Drug Alcohol Depend. 2017;174:80-90.

60. Solmi M, De Toffol M, Kim JY, et al. Balancing risks and benefits of cannabis use: umbrella review of meta-analyses of randomised controlled trials and observational studies. Bmj. 2023;382:e072348.

61. Belgers V, Röttgering JG, Douw L, et al. Cannabinoids to Improve Health-Related Quality of Life in Patients with Neurological or Oncological Disease: A Meta-Analysis. Cannabis Cannabinoid Res. 2023;8(1):41-55.

62. Pacher P, Bátkai S, Kunos G. The endocannabinoid system as an emerging target of pharmacotherapy. Pharmacological reviews. 2006;58(3):389-462.

63. McAllister SD, Abood ME, Califano J, Guzmán M. Cannabinoid cancer biology and prevention. JNCI Monographs. 2021;2021(58):99-106.

64. Pertwee R. The diverse CB1 and CB2 receptor pharmacology of three plant cannabinoids: Δ9‐tetrahydrocannabinol, cannabidiol and Δ9‐tetrahydrocannabivarin. British journal of pharmacology. 2008;153(2):199-215.

65. Abrams DI, Guzman M. Cannabis in Cancer Care. Clinical Pharmacology and Therapeutics. 2015;97(6):575-586.

66. Kleckner AS, Kleckner IR, Kamen CS, et al. Opportunities for cannabis in supportive care in cancer. Therapeutic Advances in Medical Oncology. 2019;11:1-29.

67. Mlost J, Bryk M, Starowicz K. Cannabidiol for pain treatment: focus on pharmacology and mechanism of action. International journal of molecular sciences. 2020;21(22):8870.

68. Bhattacharyya S, Morrison PD, Fusar-Poli P, et al. Opposite effects of delta-9-tetrahydrocannabinol and cannabidiol on human brain function and psychopathology. Neuropsychopharmacology. 2010;35(3):764-774.

69. D'Souza DC, Perry E, MacDougall L, et al. The psychotomimetic effects of intravenous delta-9- tetrahydrocannabinol in healthy individuals: implications for psychosis. Neuropsychopharmacology. 2004;29(8):1558-1572.

70. Knopf A. NIDA sets 5 mg of THC as standard cannabis unit for research. Alcoholism & Drug Abuse Weekly. 2021;33(19):6-7.

71. Brasky TM, Newton AM, Stephens JA, et al. Testing the feasibility of mobile ecological momentary assessment for symptom burden and management among metastatic cancer patients. Digit Health. 2024;10:doi: 10.1177/20552076241261843.

72. Kampshoff CS, Verdonck‐de Leeuw IM, van Oijen MG, Sprangers MA, Buffart LM. Ecological momentary assessments among patients with cancer: A scoping review. European Journal of Cancer Care. 2019;28(3):e13095.

73. Thong MSY, Chan RJ, van den Hurk C, et al. Going beyond (electronic) patient-reported outcomes: harnessing the benefits of smart technology and ecological momentary assessment in cancer survivorship research. Supportive Care in Cancer. 2021;29(1):7-10.

74. Moore RC, Swendsen J, Depp CA. Applications for self‐administered mobile cognitive assessments in clinical research: A systematic review. International journal of methods in psychiatric research. 2017;26(4):e1562. 75. Cui Y, Robinson JD, Rymer RE, Minnix JA, Cinciripini PM. You Don't Need an App-Conducting Mobile Smoking Research Using a Qualtrics-Based Approach. Front Digit Health. 2021;3:799468.

76. Paterson C. An ecological momentary assessment of self-management in prostate cancer survivors. Journal of Cancer Survivorship. 2019;13(3):364-373.

77. Wang Y, Jacques JJ, Li Z, Sibille KT, Cook RL. Health Outcomes among Adults Initiating Medical Cannabis for Chronic Pain: A 3-month Prospective Study Incorporating Ecological Momentary Assessment (EMA). Cannabis (Research Society on Marijuana). 2021;4(2):69.

78. Al Ghriwati N, Winter M, Semko J, Merchant TE, Crabtree VM. The feasibility and acceptability of mobile ecological momentary assessment to evaluate sleep, family functioning, and affect in patients with pediatric craniopharyngioma. J Psychosoc Oncol. 2024;42(2):159-174.

79. Ratcliff CG, Lam CY, Arun B, Valero V, Cohen L. Ecological momentary assessment of sleep, symptoms, and mood during chemotherapy for breast cancer. Psycho‐Oncology. 2014;23(11):1220- 1228. 80. Schuler T, King C, Matsveru T, et al. Wearable-Triggered Ecological Momentary Assessments Are Feasible in People With Advanced Cancer and Their Family Caregivers: Feasibility Study from an Outpatient Palliative Care Clinic at a Cancer Center. J Palliat Med. 2023;26(7):980-985.

81. Gao C, Li P, Morris CJ, et al. Actigraphy-Based Sleep Detection: Validation with Polysomnography and Comparison of Performance for Nighttime and Daytime Sleep During Simulated Shift Work. Nat Sci Sleep. 2022;14:1801-1816.

82. Chen D, Yin Z, Fang B. Measurements and status of sleep quality in patients with cancers. Support Care Cancer. 2018;26(2):405-414.

83. Grutsch JF, Wood PA, Du-Quiton J, et al. Validation of actigraphy to assess circadian organization and sleep quality in patients with advanced lung cancer. J Circadian Rhythms. 2011;9:4.

84. Brasky TM, Krok-Schoen JL, Liu J, et al. Use of Calcium Channel Blockers and Breast Cancer Risk in the Women's Health Initiative. Cancer Epidemiol Biomarkers Prev. 2017;26(8):1345-1348
